# To Be or Not to Be—Thymocyte Survival and Death in the Thymic Medulla

**DOI:** 10.3390/ijms27209078

**Published:** 2026-10-13

**Authors:** Martha C. Zúñiga

**Affiliations:** Department of Molecular, Cell, and Developmental Biology, University of California, Santa Cruz, CA 95064, USA; mzuniga@ucsc.edu

**Keywords:** thymocyte development, self-tolerance, negative selection, mimetic medullary thymic epithelial cells, CD8αβ, PD-1, kinetic proofreading, immunological synapse

## Abstract

The T cells of the mammalian immune system have a tricky challenge. They must recognize foreign antigen as “other” or “non-self” but in the context of self, while also being tolerant to the body’s own molecules and tissues. T-cell receptor (TCR) gene rearrangement occurs in the cortex of the thymus and generates receptors of relatively random specificity. The developing T cells (thymocytes) are screened in two stages, both of which are Major Histocompatibility Complex (MHC)-based. The first, positive selection, occurs in the thymic cortex where cortical thymic epithelial cells (cTECs) display self MHC + self-peptides. Thymocytes that can bind to self MHC + self-peptide then migrate to the corticomedullary junction, where they undergo negative selection. Here, they encounter antigenic peptides characteristic of peripheral tissue proteins presented by medullary antigen-presenting cells (APCs). Among the most intriguing APCs in the medulla are the mimetic medullary thymic epithelial cells (mimetic mTECs). These mimetic APCs express peripheral antigens under the direction of specific transcription factors. During negative selection, most thymocytes whose TCRs bind too well to self MHC + self-peptide die by apoptosis. However, a small proportion of autoreactive thymocytes escape negative selection and instead “alternatively” differentiate into specialized cell types that are self-tolerant and even enforce self-tolerance. The CD4 and CD8 coreceptors, which bind to MHC II and MHC I, respectively, play crucial roles in positive and negative selection. With CD8, there is an added level of complexity because there are two forms of CD8, only one of which can promote signaling through the TCR. Upon binding to MHC, CD4 and CD8 initiate signal transduction through the TCR. The strength of signal transduction through the TCR and through co-stimulatory CD28 determines the outcome of TCR-MHC + peptide binding. Signal transduction occurs within the immunological synapse and is regulated by kinetic proofreading mechanisms. In summary, negative selection involves interactions between developing thymocytes and a variety of medullary APCs. These molecular interactions occur at specialized membrane domains that bring together the components of the TCR signaling apparatus, which are subject to kinetic proofreading. Together, the regulatory mechanisms occurring in developing thymocytes and in medullary APCs generate a T-cell arsenal that is tolerant of self and active against foreign entities.

## 1. Introduction—In the Beginning

Vertebrate animals respond to an ever-changing antigenic universe by having an adaptive immune system. It is adaptive in two ways. Firstly, its constituent B cells and T cells are generated daily. Secondly, these lymphocytes collectively express a diverse set of antigen receptors of different specificities. These unique antigen specificities are embodied in antigen binding sites that are created by seemingly random gene rearrangement mechanisms that occur during B-cell and T-cell development. Consequently, it is possible for a B cell or T cell to express receptors that have affinity for self-molecules. To counteract this danger, during their development, both B cells and T cells undergo selection processes that eliminate cells bearing autoreactive receptors. In the case of T cells expressing an αβT-cell receptor for antigen, (αβTCR), this occurs in the thymus via a two-step process. Both steps rely on the fact that the ligand for the αβTCR is an MHC molecule bearing an antigenic peptide. These two steps are called positive and negative selection. In this review, I focus on negative selection. I discuss findings from the immunology literature on negative selection events and mechanisms, which occur at the thymic corticomedullary junction and in the medulla. While I discuss both the fates of thymocytes bearing TCRs specific for MHC II + peptide and MHC I + peptide, my discourse on events occurring in thymocytes bearing MHC I-restricted αβTCRs is more extensive. Finally, I discuss fascinating events occurring in medullary APCs that determine the fate of thymocytes bearing autoreactive αβTCRs and that function to enforce self-tolerance.

## 2. First and Foremost, Know Who You Are

**Figure 1:**
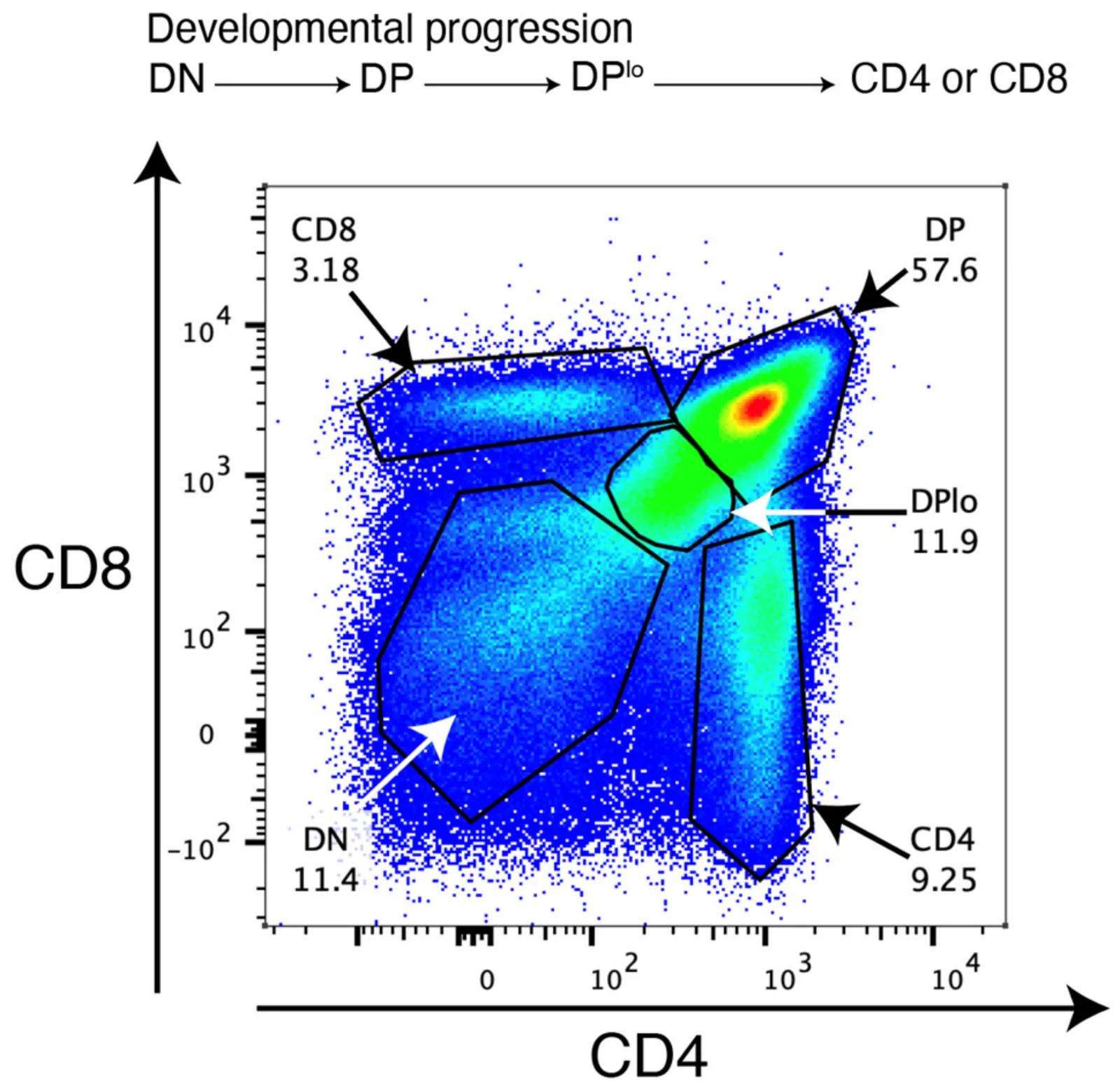
CD4 and CD8 track the developmental progression of thymocytes expressing αβTCRs. Shown is a flow cytometry plot of mouse thymocyte populations as identified by expression of CD4 and CD8. Above the flow cytometry plot are indicated the five major populations of developing thymocytes (DN, DP, DP^lo^, CD4, and CD8) and their occurrence during thymocyte developmental progression.

T-cell development occurs in the thymus and begins in the thymic cortex. As stated above, TCR gene rearrangement events in developing thymocytes generate a diverse array of antigen receptors, each with its unique specificity. The gene rearrangement process involves several steps [1]. There are two kinds of TCRs—an αβTCR and a γδTCR. Only αβTCRs recognize MHC/peptide complexes, and so all subsequent discussion in this review focuses on thymocytes expressing αβTCRs. In addition to the αβTCR, either of two coreceptors—CD4 or CD8—also bind to MHC. These two coreceptors are distinguished by their MHC specificity. CD4 is specific for MHC II, and CD8 binds MHC I. CD4 and CD8 are of paramount importance in positive and negative selection in two ways. Firstly, they increase the avidity of αβTCR binding to its cognate MHC + peptide ligand. Secondly, they help initiate signal transduction events that lead to the survival of a thymocyte or to its death. Prior to TCR gene rearrangement, thymocytes express neither CD4 nor CD8 and are said to be double negative (DN, Figure 1). Upon the generation of a properly formed TCR, the thymocytes express both CD4 and CD8 and are said to be double-positive (DP). Two important events happen at the DP stage of thymocyte development: positive selection and lineage commitment, which I will discuss shortly.

Lineage commitment is the developmental decision to become either a CD4^+^ helper T cell or a CD8^+^ cytotoxic T cell [2,3]. Until recently, it was believed that signaling through either CD4 or CD8 determines a thymocyte’s lineage choice. However, results from single-cell studies on DP thymocytes showed that engagement of CD4 or CD8 by MHC is not the sole determinant of a thymocyte’s lineage fate [4]. During positive selection, cis-regulatory elements in the *cd4* and *cd8* genes regulate both the kinetics of the expression of these co-receptor genes and the duration of TCR signaling [5]. Concomitantly, the developing thymocyte commits to its helper or cytotoxic function. The master transcriptional regulators—ThPOK (in the case of CD4) [6,7] and Runx3 (in the case of CD8) [8,9,10,11]—are required both for commitment to expression of either CD4 or CD8 and for lineage function. Robey and her colleagues have provided a comprehensive review detailing the intricacies of the molecular regulation of *cd4* and *cd8* transcription [12].

Also important during thymocyte development is the co-stimulatory molecule CD28. CD28 is a cell-surface glycoprotein that is expressed by mature T cells, where it plays a critical role in T cell activation [13]. CD28’s value stems from the fact that its binding to its ligand—CD80 or CD86—amplifies signaling through the TCR [14,15,16,17,18,19]. In mature T cells in peripheral lymphoid tissues, the binding of CD28 to either CD80 or CD86 on APCs delivers a second signal that is required for T-cell activation [20]. This is particularly crucial when low numbers of TCRs are engaged or when the TCRs have weak affinity for their MHC + peptide ligands [21]. Not surprisingly, CD28 is also found on thymocytes. DP thymocytes, DP^lo^ thymocytes (which are developmentally downstream of DP thymocytes; Figure 1), and CD4^+^ and CD8^+^ single positive (SP) thymocytes all express CD28 [22]. However, in the thymus, CD80 and CD86 are chiefly expressed by thymic medullary APCs [23,24] and hence, the CD28 co-stimulatory signal is chiefly operative during negative selection.

## 3. Growing up Is Hard to Do

**Figure 2:**
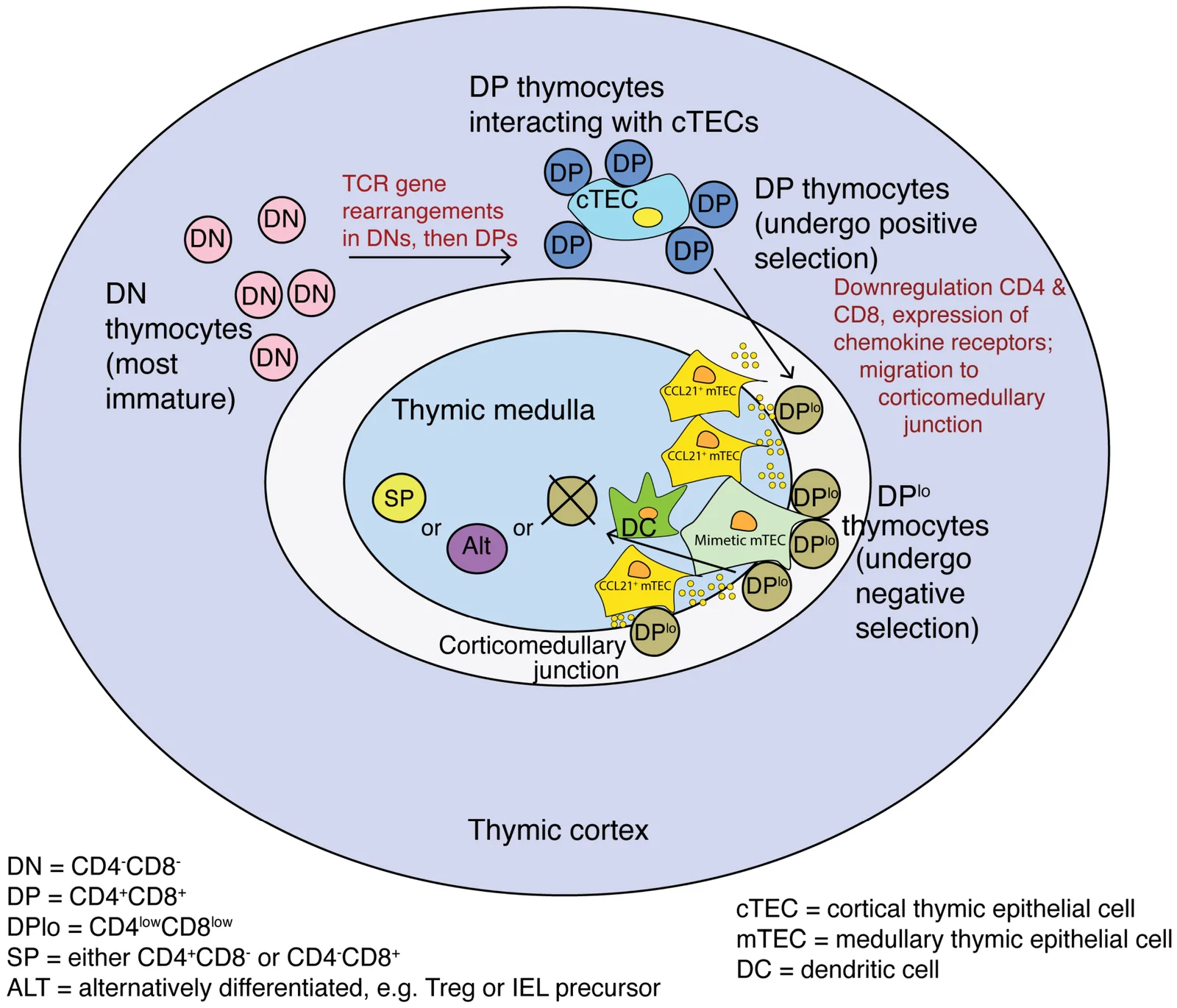
Cellular interactions and locations of thymocyte development. For clarity purposes, molecules, such as TCR, CD4, CD8, CCR7, CCL21, etc., are not shown. These details are within the text. Small yellow circles represent secreted CCL21. The cell with a large X across it is an apoptotic cell.

The steps in thymocyte development and the thymic locations in which these steps occur are outlined briefly in Figure 2. As stated earlier, CD4’s and CD8’s roles in thymocyte development begin at the DP stage. Central to thymocyte development is the binding of αβTCRs on thymocytes to MHC+ peptide complexes occurring on the surfaces of thymic epithelial cells (TECs) and other thymic stromal cells, such as dendritic cells (DCs). During interactions with cortical TECs (cTECs), a CD4^+^CD8^+^ DP thymocyte that can bind its TCR’s cognate MHC + peptide ligand is positively selected for survival. After positive selection, expression of CD4 and CD8 diminish. The downregulation is not equivalent for both coreceptors [12]. However, for simplicity’s sake, I will refer to this stage as the DP^lo^ thymocyte. The DP^lo^ thymocyte expresses the chemokine receptor, CCR7, which enables it to migrate towards the chemokine CCL21. Since CCL21 is produced by thymic medullary cells, the DP^lo^ thymocyte migrates to the corticomedullary junction [25]. (Interestingly, when migration to the medulla is prevented, as in the case of mice deficient both in CCL21 and in CCR7, SP thymocytes still develop, but they accumulate in the thymic cortex [26,27]).

**Figure 3:**
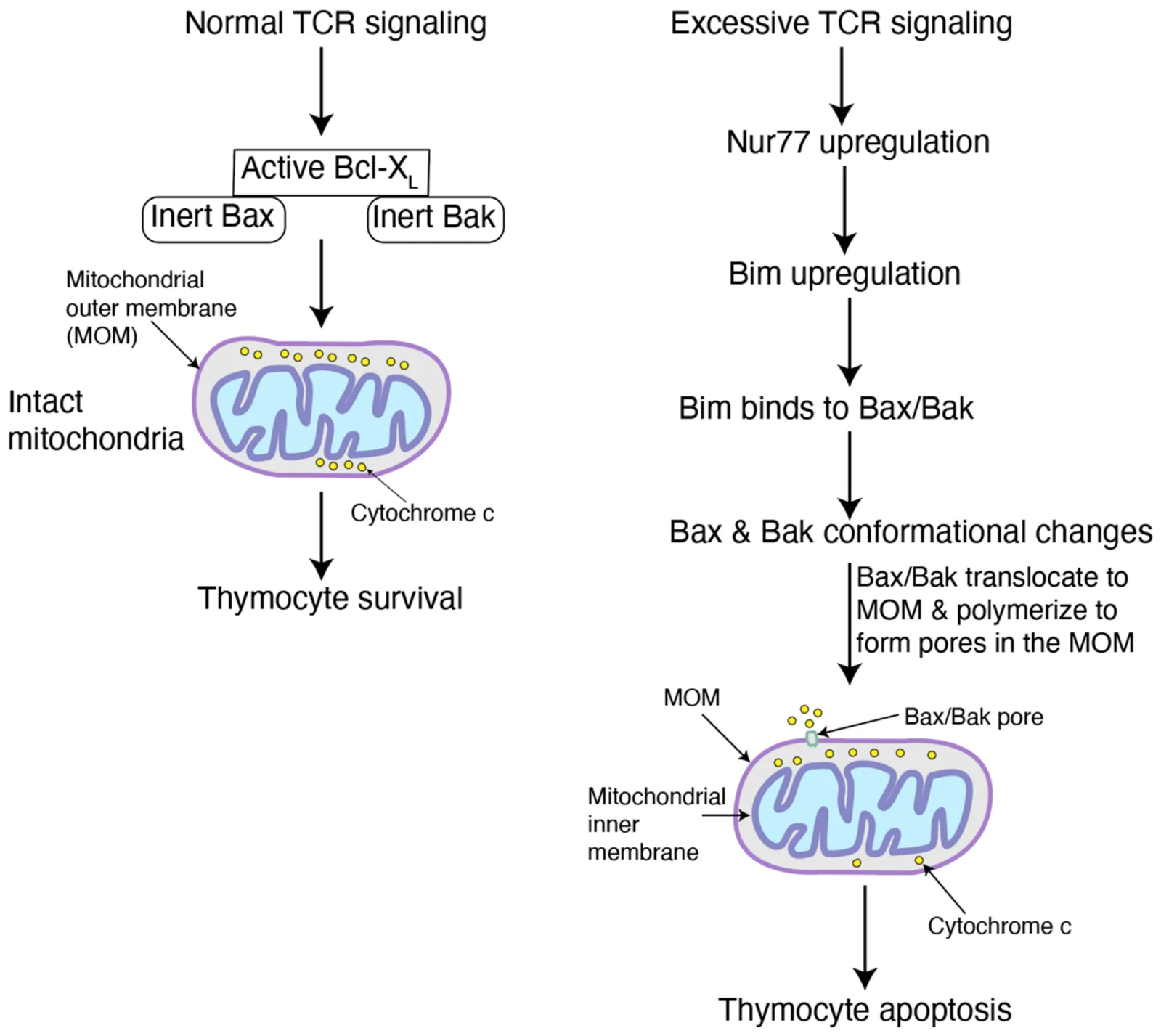
The intrinsic or mitochondrial apoptotic pathway. Members of the Bcl-2 protein family control entry into the intrinsic apoptotic pathway. Apoptosis is dependent on permeabilization of the mitochondrial outer membrane (MOM). As discussed in the text, following Bax- or Bak-mediated pore formation, cytochrome c and other components lead out of the mitochondrion. In the cytosol, they activate the caspase cascade, leading to cell death.

At the corticomedullary junction, the DP^lo^ thymocyte interacts with medullary TECs (mTECs) and other medullary APCs such as medullary DCs (mDCs). If the DP^lo^ thymocyte undergoes moderate signaling through its TCR, it becomes a CD4^+^ or CD8^+^ SP thymocyte and migrates out to peripheral lymphoid tissues. Conversely, strong signal transduction through the TCR leads to death via apoptosis (Figure 3) or to divergent differentiation, as follows: A thymocyte bearing an MHC II-specific TCR that survives negative selection differentiates into a CD4^+^ regulatory T cell via a Treg precursor (Treg; Table 1) [28,29,30]. A thymocyte bearing an MHC I-specific TCR that escapes negative selection can become an intraepithelial lymphocyte precursor (IEL precursor; Table 1). However, as described below, not all MHC I-specific thymocytes that survive negative selection become IEL precursors.

## 4. Suicide Is Painless. It Brings on Many Changes—The Orchestrated Events of Apoptosis

During negative selection, thymocytes that bind too well to self MHC + self-peptide die via the intrinsic pathway of apoptosis. This pathway is regulated by members of the Bcl-2 family. In healthy cells, the pro-apoptotic effectors, Bax and Bak, occur in the cytosol in an inert form (Figure 3). Strong signaling through the TCR causes the upregulation of BH3-only members of the Bcl-2 family, including Bim and Bak. This upregulation is mediated by the Nr4a nuclear hormone receptor Nur77 (Figure 3). Indeed, Nur77 is required for the upregulation of Bim in thymocytes expressing an autoreactive TCR [34]. BH3-only proteins, such as Bim, then bind to Bax or Bak, inducing a conformational change that leads to their oligomerization [35]. The oligomerization and insertion into the mitochondrial outer membrane (MOM) occur in steps [36] and result in pore formation and release of cytochrome c. Once in the cytosol, cytochrome c binds to apoptotic protease-activating factor-1 (Apaf-1), leading to the formation of a cytosolic multi-protein complex called the apoptosome [37]. The apoptosome recruits inactive caspase-9, leading to its activation, which leads to downstream proteolytic events that culminate in apoptosis [38,39]. Bim also promotes apoptosis by inhibiting the pro-survival protein Bcl-XL, presumably by sequestering it [40].

Gene knockout studies have demonstrated a requirement for Bim for the deletion of thymocytes bearing autoreactive TCRs [41] and specifically during negative selection [42]. Loss of both Bim and Puma allowed the escape from apoptosis of a significantly greater number of autoreactive thymocytes [43]. However, unlike Bax and Bak, Puma does not form pores in the MOM. Instead, Puma binds with high affinity to Bcl-XL and Bcl-2, thereby freeing Bax and Bak to oligomerize and to form pores in the MOM [44]. Puma also stabilizes Bax/Bak in a conformation that enables that polymerization [44].

## 5. With a Little Help from My Friends

**Figure 4:**
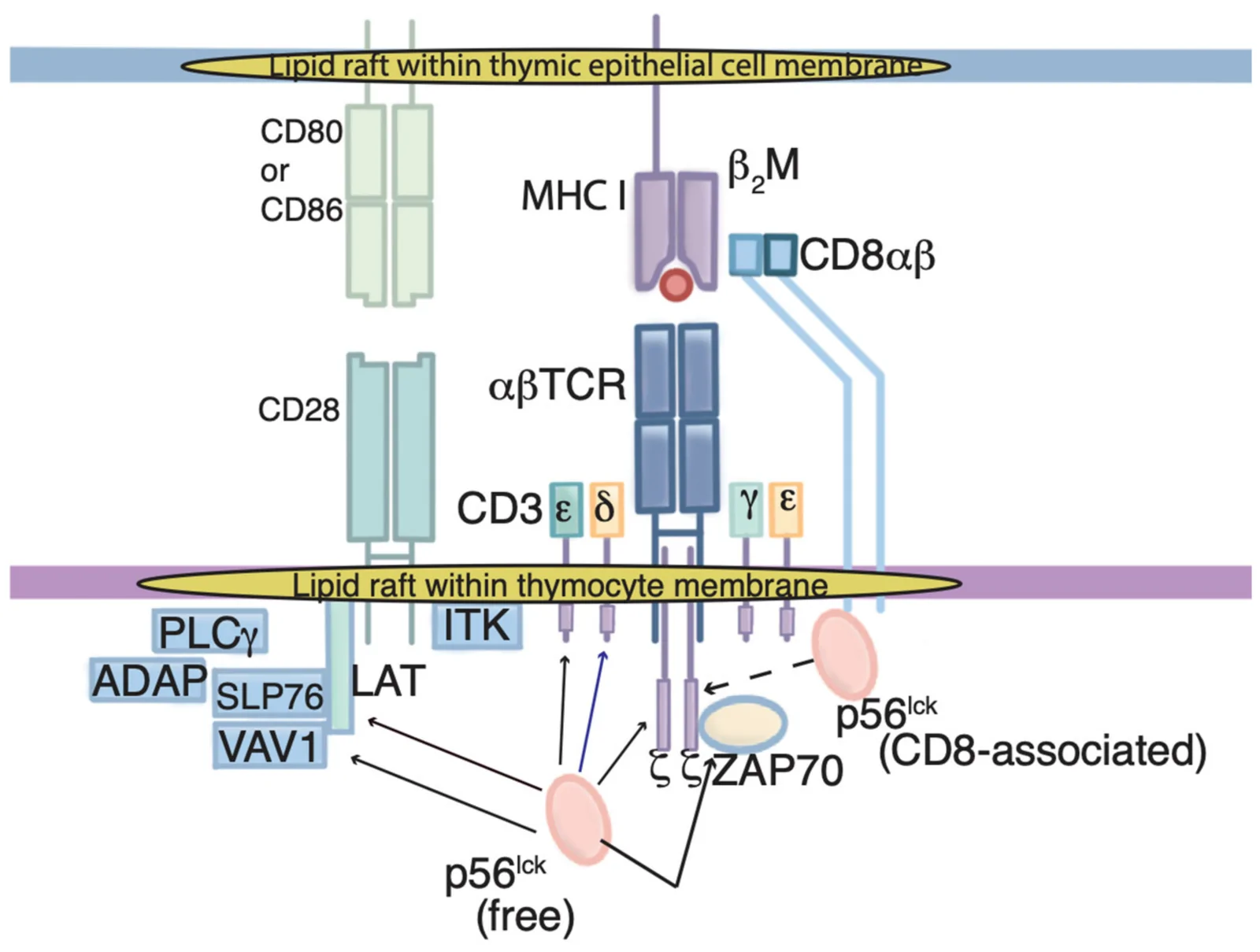
Signal transduction events required for activation via the TCR occur within the lipid raft that constitutes the immunological synapse. As discussed in the text, binding of CD8αβ to MHC I (illustrated here) or of CD4 binding to MHC II launches a series of phosphorylation events that promote signal transduction through the TCR. The first step in the signal transduction cascade is the phosphorylation of the CD3ζ ITAMs by p56^lck^. p56^lck^ can occur both freely in the cytoplasm and bound to the coreceptor (in this case, CD8αβ). Arrows identify phosphorylation targets. Further details are provided in the text. Not shown are the adhesion molecules’ lymphocyte function-associated antigen 1 (LFA-1, an integrin) [52] on the thymocyte membrane and its ligand, Intercellular Adhesion Molecule I (ICAM-1) [53] on the TEC membrane.

The TCR is a complex of several polypeptides. In the case of the αβTCR, the antigen-binding properties reside in the assembled α and β chains of the TCR. These chains have no signaling capabilities and are associated with the CD3 complex of polypeptides, which are required for signal transduction (Figure 4). The ζ chains of the CD3 complex have long cytoplasmic tails that contain immunoreceptor tyrosine-based activation motifs (ITAMs), which can be phosphorylated by tyrosine kinases. This is where CD4 and CD8 come in. CD4 and CD8 have both extracellular and intracellular moieties and hence, dual functions. Via their extracellular domains, CD4 and CD8 bind to MHC II and MHC I, respectively, and via their cytoplasmic tails, they bind to a tyrosine protein kinase called p56^lck^ [45]. During the binding of MHC + peptide, the αβTCR, CD4, CD8, and other associated molecules cluster at the immunological synapse, which is the junction between the T cell (or thymocyte) and the APC. The coordinated engagement of MHC by both the αβTCR and by CD4 or CD8 launches an enzymatically based signal transduction cascade [46]. Specifically, the binding of CD8 or CD4 and of αβTCR to MHC + peptide facilitates the phosphorylation of the ITAMs of CD3ζ by p56^lck^ [46] (Figure 4). p56^lck^ also phosphorylates Zeta-chain-associated protein kinase 70 (ZAP-70). ZAP-70 then phosphorylates the adaptor proteins, Linker for activation of T cells (LAT) and SLP-76 [47,48,49] (Figure 4). These proteins are joined in the immunological synapse by Protein kinase Cθ [50] and phospholipase Cγ (PLCγ) [51] (Figure 4). This completes the assemblage of the collection of signal transduction complexes (the TCR signalosome) required for efficient T cell activation.

Since CD4 is a single-chain coreceptor, all the essential features for ligand binding and signal transduction are vested in the single CD4 polypeptide chain. The situation is more complicated for CD8, which is a dimer that can occur in either of two forms [54]. All T cells (except for IELs) express CD8 as a heterodimer of CD8α and CD8β. IELs express CD8 as a CD8αα homodimer. CD8αβ is much more effective in T-cell activation than is CD8αα [55,56]. This was initially surprising because CD8α can bind to p56^lck^ through its cytoplasmic tail [57] and thus, should be able to promote signal transduction. The signaling prowess of CD8αβ and hence, its efficacy as a coreceptor for the TCR, stem from properties that are unique to CD8β. CD8β promotes the interaction of CD8αβ with the αβTCR [49], thereby facilitating the phosphorylation of CD3ζ-chain ITAMs (Figure 4). Also, via CD8β, CD8αβ efficiently recruits ZAP-70, thereby enabling its phosphorylation and activation by p56^lck^ [47,48,49].

In DP and DP^lo^ thymocytes, CD8αβ must compete with CD4 for binding to p56^lck^. CD4 has higher affinity for p56^lck^ than does CD8αβ [45]. Hence, in thymocytes expressing both CD4 and CD8αβ (i.e., DP and DP^lo^ thymocytes), the number of p56^lck^-associated CD8αβ molecules is lower than is the number of p56^lck^-associated CD4 molecules [58]. This discrepancy is important to consider in the context of negative selection for the following reasons. Any given MHC molecule can bind a diversity of peptides. Therefore, only a small fraction of the MHC + peptide complexes on the surface of a thymic TEC is expected to be bound by a particular αβTCR. Consequently, the MHC/antigenic peptide combination that is a suitable ligand for any given MHC I-specific αβTCR on a developing DP^lo^ thymocyte may be present at suboptimal levels on the surface of the TEC. In this situation, a diminished number of p56^lck^-associated CD8αβ molecules in the immunological synapse could result in inefficient signaling and failure to undergo negative selection.

As mentioned previously, CD28 binding to CD80 or CD86 amplifies signaling through the αβTCR. However, CD28 seems not to be equally important for negative selection of all thymocytes. CD28 interaction with CD80/CD86 on mTECs invariably promotes either apoptosis of a DP^lo^ thymocyte expressing MHC II-specific αβTCRs or its development into a CD4^+^ Treg [59]. By contrast, in the case of DP^lo^ thymocytes expressing MHC I-specific αβTCRs, the need for CD28 during negative selection appears to vary with the affinity or avidity of the αβTCR for its MHC I/peptide ligand [60].

## 6. Location, Location, Location—Lipid Rafts and the Immunological Synapse

The first step in the activation of a T cell involves the binding of the αβTCR and of the relevant coreceptor (CD4 or CD8) to MHC + peptide ligand, of CD28 to CD80 or CD86 (Figure 4), and of other molecules (such as the adhesion molecule, LFA-1) to their own ligands. As noted earlier, these interactions occur within the immunological synapse [61]. Integral to the clustering of molecules within the immunological synapse are lipid rafts [62]. Lipid rafts are dynamically changing assemblages of cholesterol and sphingolipids [63]. A variety of studies indicate that lipid raft-mediated interactions are central to TCR signaling [64,65,66,67]. Associations of T-cell proteins with their ligands on APCs stimulate the lateral mobility of the molecules within the plasma membrane and into lipid rafts. Also, the various proteins on the membranes of the T cell (or thymocyte) and the APC influence each other. For example, αβTCR localization into lipid rafts is promoted by CD28 binding to CD80 or CD86 during T cell activation [68]. This results in the clustering of the αβTCR and other relevant proteins into a supramolecular protein complex (or signalosome) within the immunological synapse (Figure 4).

Both CD4 and CD8αβ migrate into lipid rafts. However, CD8β—but not CD8α—can promote the movement of CD8αβ into lipid rafts [69]. Palmitoylation of the CD8β cytoplasmic tail enables CD8αβ to partition into lipid rafts [70,71], but this is not the sole determinant of lipid raft localization. In the case of human CD8αβ, an arginine motif in the CD8β cytoplasmic region also contributes to lipid raft localization [69]. The ability of CD8β to promote CD8αβ partitioning into lipid rafts helps to explain the requirement for the CD8β chain for positive selection of MHC I-specific thymocytes [72,73,74]. As discussed further in Section 8, the abundance of CD8β, and therefore of cell-surface CD8αβ, also is essential for the negative selection of thymocytes bearing self-reactive MHC I-specific αβTCRs [75,76].

Just as membrane domains, such as lipid rafts are thought to self-organize during signaling, cytoplasmic compartmentalization also occurs. Specifically, biomolecular condensates form through weak but multivalent macromolecular associations [77]. These biomolecular condensates can promote clustering of receptors on the plasma membrane [78]. Vale and colleagues biochemically reconstituted 12 components of the TCR signaling pathway onto model membranes in the presence of actin [79]. In this system, phosphorylation of TCR CD3 chains caused the spontaneous formation of biomolecular condensate clusters of LAT. Furthermore, both in an *in vitro* lipid bilayer model and in human Jurkat T cells, LAT clustering promoted actin polymerization [79]. Studies with giant plasma membrane vesicles from activated Jurkat T cells showed coincident localization of cholesterol with condensates of LAT and the adaptor protein Grb2 [80], which docks onto phosphotyrosines on LAT. From these and other studies emerges a model in which T-cell activation induces the formation of cytoplasmic protein condensates that associate with and stabilize lipid rafts. At the same time, membrane lipid rafts stabilize LAT condensates [80]. Thus, signalosome formation in lipid rafts appears to promote the efficient assembly of cytoplasmic signaling molecules into biomolecular condensates. I will return to lipid rafts in Section 10.

## 7. Decisions, Decisions, Decisions: Kinetic Proofreading’s Role in a Thymocyte’s Fate

Proximity is not the sole determinant of the outcome of TCR binding to MHC + peptide. McKeithan [81] articulated a kinetic proofreading model in which quantitative differences in signal strength dictate the outcome of antigen binding. Specifically, he noted that the binding of MHC + peptide by the TCR (and of CD4 or CD8 also binding to MHC) initiates a series of phosphorylation events in downstream signaling components [81]. He proposed that the multiplicity of these phosphorylation events enforces a time delay between the initiating binding of the TCR to its MHC + peptide ligand and eventual T cell activation [81,82]. McKeithan also proposed a nonequilibrium situation in which differences in aggregate sizes afford a type of kinetic proofreading in that small clusters and larger clusters generate distinct signals. These clusters could be organized in lipid rafts. Different cluster sizes in lipid rafts could yield quantitative differences in signaling strength and thus, distinctly different outcomes for autoreactive thymocytes during negative selection.

Within signaling clusters, intra- and inter-molecular events provide additional proofreading opportunities. Here, I briefly discuss two examples. ZAP-70 consists of an N-terminal regulatory module and a C-terminal kinase domain that has catalytic function. The regulatory domain of ZAP-70 consists of tandem Src homology-2 (SH2) repeats [83]. Biophysical and biochemical studies suggest that allosteric interactions of the SH2-binding domains regulate signaling via the TCR [84]. These allosteric interactions may provide a means for kinetic proofreading. Another key proofreading node involves LAT. Slow phosphorylation of Y_132_ in LAT has been proposed to be the most important rate-limiting step in signaling via the TCR after MHC + peptide binding [82]. These examples emphasize the complex ways in which T-cell signaling cascades influence the ultimate consequence of αβTCR binding to its MHC + peptide ligand.

## 8. Slipping Under the Radar

In this section of the review, I will focus on the role of CD8αβ in negative selection. As already stated, the prevailing view is that thymocytes bearing αβTCRs that have high affinity for self MHC I/peptide either die by apoptosis or develop into IEL precursors [32,85,86,87]. In unmanipulated mice, there are ~5 million CD8αα^+^ IELs bearing autoreactive αβTCRs [88]. How does signaling strength determine if an autoreactive thymocyte dies by apoptosis or becomes an IEL precursor? One study showed that CD8αα^+^ IELs can develop from DP^lo^ thymocytes when αβTCR engagement occurs in the absence of CD28 co-stimulation [88]. This result is surprising since CD28 signaling is thought to amplify signaling through the TCR. Moreover, it is not clear how this result can be explained in a kinetic proofreading context.

Since the CD4 and CD8 coreceptors bind to MHC to initiate signal transduction via the TCR, they are expected to contribute to signal transduction strength. Robey and her colleagues used a thymic tissue slice experimental system to study in real time the development of thymocytes bearing an MHC I-specific αβTCR [89]. Their experimental model was the OT-I TCR transgenic mouse (which expresses an αβTCR that is specific for H-2K^b^ + chicken ovalbumin peptide SIINFEKL). They laid OT-I thymocytes upon thymic slices bathed in SIINFEKL peptide and assessed thymocyte development over time. In this system, DP thymocytes downregulated both CD4 and CD8, consistent with findings from previous studies [90]. Some of these DP^lo^ thymocytes survived negative selection and became IEL precursors. In the surviving IEL precursors, CD8β levels remained low, but CD8α levels did not [89]. Hence, these thymocytes were largely CD8αα^+^.

The low CD8β level of the surviving DP^lo^ thymocytes in these studies brings front and center the role of CD8αβ in signal transduction within the immunological synapse. Specifically, since CD8αβ (but not CD8αα) can partition into lipid rafts, DP^lo^ thymocytes expressing autoreactive αβTCRs and having *insufficient* numbers of CD8αβ dimers might escape negative selection. My colleagues and I employed a probabilistic gating procedure to examine this possibility in a transgenic mouse αβTCR model [33]. This double transgenic model employs CBA (H-2^k^) mice expressing allogeneic H-2K^b^ in the thymic medulla and an anti-H-2K^b^ TCR. We found that the viability of double transgenic DP^lo^ thymocytes remained high across a range of CD8β/α ratios but then dropped precipitously at a specific CD8β/α ratio [33]. We inferred that in DP^lo^ thymocytes having low CD8β/α ratios, there is a shortage of p56^lck^-associated CD8αβ molecules in lipid rafts (and hence, in the immunological synapse). We further inferred that this deficiency of p56^lck^-associated CD8αβ results in inadequate signaling through the anti-H-2K^b^ TCR, thereby allowing escape from negative selection.

## 9. Wait—There’s More, and It’s Complicated!

Programmed cell death protein 1 (PD-1) is a cell-surface protein, which upon binding to its molecular ligand—either PD-L1 or PD-L2—on APCs, attenuates T-cell activation [91,92]. PD-1 does this by recruiting SHP-2 phosphatase to its cytoplasmic tail [93]. This results in the suppression of phosphorylation both of CD3 chains and of ZAP-70 [93]. Thus, in mature T cells, PD-1 plays a key role in regulating signal transduction through the αβTCR. PD-1 also is expressed in the thymus, where it plays a regulatory role during positive selection [94]. But does PD-1 have a role during negative selection? In their thymic slice experiments, the Robey group observed a slight increase in PD-1 expression in OT-I DP^lo^ thymocytes that had been exposed to SIINFEKL peptide [89]. This result was consistent with earlier studies in non-transgenic mice showing that “developmentally diverted” thymocytes that had survived negative selection have elevated levels of PD-1 [88]. However, PD-1 expression is not required for developmental diversion since it occurred in mice in which the *pd1* gene had been deleted [88]. Nevertheless, PD-1 may promote the survival of thymocytes expressing a self-reactive αβTCR during negative selection by attenuating signaling through the αβTCR [93,95,96,97]. We examined this possibility in our studies. We found that the importance of PD-1 for the survival of autoreactive DP^lo^ thymocytes was conditional [33]. Specifically, PD-1 was important only in autoreactive DP^lo^ thymocytes having *intermediate* levels of CD8β. At very low levels of CD8β (and therefore low levels of CD8αβ), DP^lo^ thymocytes bearing a self-reactive αβTCR survived, regardless of PD-1 levels. Conversely, DP^lo^ thymocytes bearing a self-reactive αβTCR and having high CD8β levels (and therefore high CD8αβ levels) were apoptotic, even when PD-1 levels were high [33].

## 10. Let Me in—Lipid Rafts Revisited

**Figure 5:**
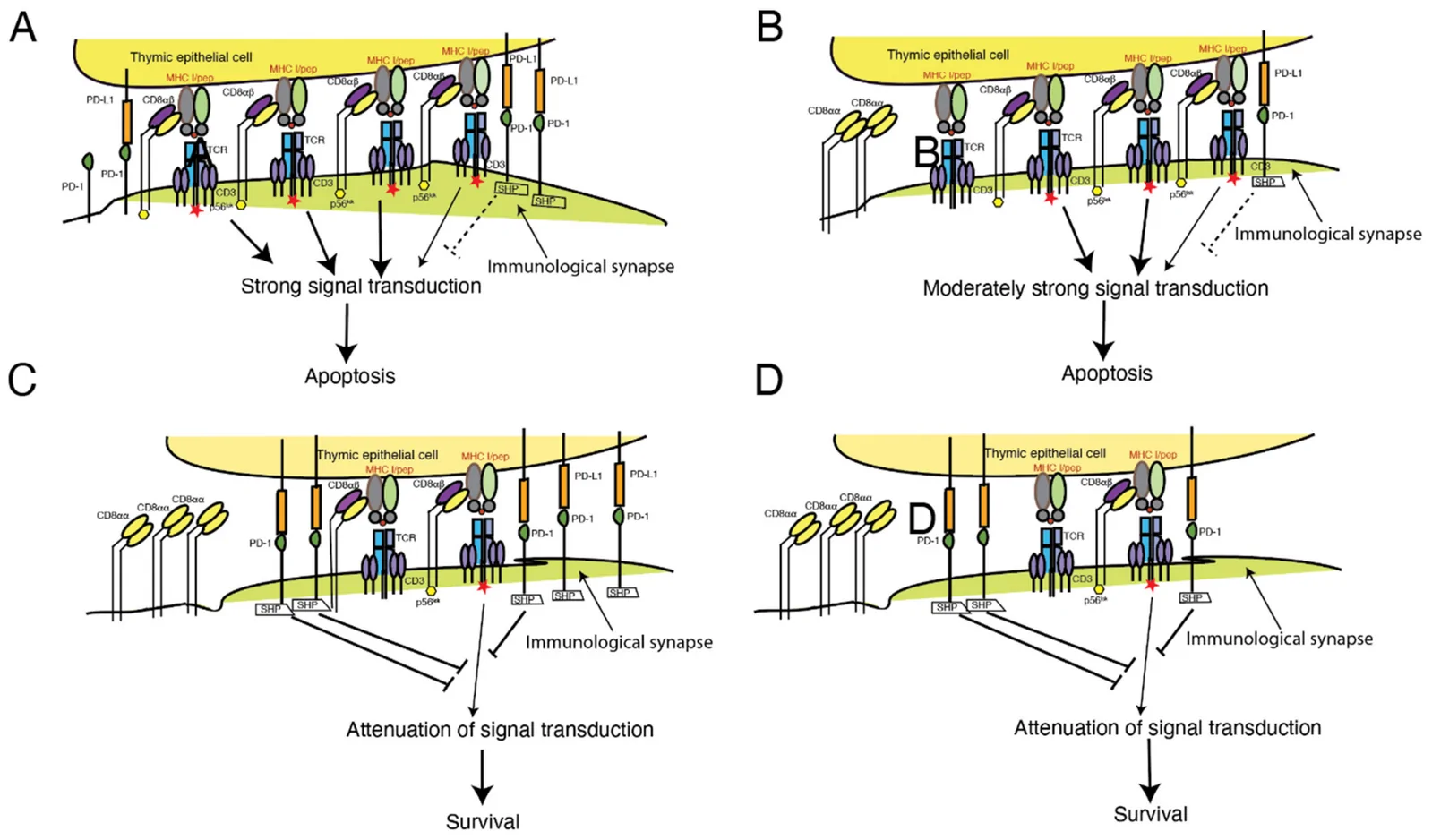
Roles of CD8αβ and PD-1 in negative selection. Panel (**A**) A high density of CD8αβ promotes strong signal transduction through the αβTCR, resulting in the apoptosis of autoreactive post-selection DP^lo^ thymocytes, regardless of PD-1 levels. Panel (**B**) When there is not enough PD-1 to attenuate signaling, even moderate levels of CD8αβ sufficiently augment signaling through the αβTCR to promote apoptosis. Panel (**C**) Autoreactive thymocytes can survive when PD-1 levels are high and CD8αβ levels are low to moderate. Panel (**D**) Autoreactive thymocytes can survive when CD8αβ levels are very low regardless of the PD-1 level. The red star is an indication of phosphorylation of CD3 ITAMs. The yellow hexagon is p56^lck^. Blunt-ended arrows indicate blocking of the indicated step. Lighter arrows and dashed blocked arrows indicate a lesser effect.

As discussed earlier in Section 6, the immunological synapse is thought to be a staging ground for formation of the TCR signalosome. For PD-1 to interfere with TCR signaling, it must gain proximity to the TCR signalosome. A recent study reported that PD-1 is recruited into lipid rafts by CD48 [98]. If both CD8αβ and PD-1 can enter lipid rafts (and thus, the immunological synapse), then what determines if CD8αβ promotes signal transduction or if PD-1 squelches it? It occurred to us that the answer might lie in the small size of lipid rafts [99]. It has been estimated that no more than 10–30 proteins can occur in a lipid raft [99,100]. We thus hypothesized that CD8β is more effective in targeting CD8αβ to lipid rafts than CD48 is in recruiting PD-1 to them [33]. If this is true, then when CD8αβ is abundant, it might outcompete PD-1 for space in the lipid rafts of the immunological synapse. In this hypothetical scenario, there wouldn’t be enough PD-1 to suppress signal transduction through the αβTCR, and the strong signaling would lead to cell death by apoptosis. The predictions of this working model are illustrated in Figure 5, in which four different scenarios are depicted.

Our working model is consistent with studies on the formation of microclusters in the membranes of activated T cells. Microscopically observed microclusters are not synonymous with lipid rafts. Nevertheless, there is reason to believe that microclusters contain lipid rafts. In *in vitro* studies with T cell lines and mature CD4^+^ T cells), PD-1 microclusters and TCR microclusters co-localized upon the binding of PD-1 to PD-L1 or PD-L2 [96,101]. Functional studies indicated that colocalization of the TCR and PD-1 microclusters is required for effective suppression of TCR signal transduction [96]. PD-1 microclusters were found to form in PD-1^hi^ OT-I TCR^+^ transgenic CD8^+^ T cells but not in naïve OT-I TCR^+^ transgenic CD8^+^ T cells, which don’t have high levels of PD-1. The implication of this observation is that high levels of PD-1 are required for PD-1 to enter TCR microclusters. This idea is consistent with the basic premise of our working model.

PD-1 binds to the Src homology 2 domain-containing phosphatase 2 (SHP-2) [102], a member of the non-receptor protein-tyrosine phosphatase family. It has been suggested that PD-1 sequesters SHP-2 so that it cannot remove inhibitory phosphate groups from p56^lck^ [46]. Furthermore, SHP-2 phosphatase recruited by PD-1 dephosphorylates the CD3ζ chains but it dephosphorylates CD28 more efficiently than it does CD3ζ [96,101]. Thus, recruitment of SHP-2 to the TCR signalosome is critical for PD-1-mediated attenuation of TCR signaling.

SHP-2 (but not SHP-1) was observed to be recruited to PD-1 microclusters [96,101]. Studies with PD-1 chimeras having varying sizes of the extracellular domains showed that PD-1 molecules with larger extracellular domains did not co-localize in clusters with TCRs and failed to abrogate TCR signaling [96]. The larger PD-1 molecules were apparently excluded from the microclusters. These studies show that PD-1 proximity to the TCR signalosome is essential for SHP-2 recruitment to TCR microclusters and for SHP-2-mediaated inhibition of signaling. This requirement for proximity suggests the plausibility of our working model.

Unfortunately, it will be difficult to test our hypothetical model. Lipid rafts are too small to be observed with standard light microscopic techniques. Antibodies and/or lectins must be used to promote clustering of lipid raft components to detect them microscopically [103,104]. Hence, it may be difficult to definitively test this hypothesis in a physiologically relevant experiment. The assembly of T-cell receptor components into multimeric signaling complexes should increase residence time in lipid rafts. Nevertheless, different proteins may partition into lipid rafts with different kinetics and thus have different residence time in them.

## 11. A Simple Twist of Fate

Intraepithelial lymphocytes (IELs) [32,88,105,106] are derived from IEL precursors (IELps), which themselves develop from CD5^+^αβTCR^+^ thymocytes. There are two thymic IELps [31,32]. The major IEL population is called type A. Type A IELps arise during negative selection and express PD-1 [32]. The minor population (type B IELps) are PD-1^−^ and Tbet^+^. In mice, type B IELps leave the thymus earlier in life than do type A IELps, which are more prevalent in adults [32].

Several research groups have demonstrated beautifully that in adult mice, MHC I-restricted CD5^+^DP^lo^ thymocytes that survive negative selection become IEL precursors and go on to populate the intestine [32,85,86,87,88,89]. The implication—and in some minds, the assumption—has been that for a thymocyte bearing a TCR specific for self MHC I + self-peptide, the only alternative to apoptosis is to become an IEL precursor. Is this in fact true? Unlike thymocytes destined for peripheral lymphoid tissues [26], type A IEL precursors do not express CCR7 [32]. Our studies demonstrated that in double-transgenic mice that express both allogeneic H-2K^b^ in the medulla and the anti-H-2K^b^ TCR, surviving DP^lo^ post-selection thymocytes give rise to two types of DN thymocytes. These are PD-1^+^DN thymocytes that express CCR7 and PD-1^+^ DN thymocytes that do not express CCR7 [33]. Furthermore, we found transgenic αβTCR^+^ DN lymphocytes in the peripheral lymph nodes of these mice [33]. These DN lymphocytes are presumed to be derived from αβTCR^+^PD-1^+^CCR7^+^DN thymocytes since thymocytes destined for peripheral lymph nodes express CCR7. These results suggest that surviving thymocytes bearing autoreactive TCRs are not necessarily restricted to becoming IELs but also give rise to lymphocytes that populate other peripheral lymphoid tissues.

## 12. Live and Let Live—Becoming an Enforcer of Self-Tolerance

It is important to remember that the vast majority of thymocytes expressing self-reactive TCRs die during negative selection. To prevent autoimmunity, the surviving thymocytes must be restrained. The immune system has distinct ways of enforcing self-tolerance in the small proportion of potentially autoreactive CD4^+^ and CD8^+^ T cells that escape negative selection. As stated previously, surviving thymocytes bearing autoreactive MHC II-specific TCRs become CD4^+^ Treg cells that enforce tolerance to self [28,29,30]. In the case of the CD8 lineage, thymocytes bearing autoreactive MHC I-specific TCRs give rise to CD8αα^+^ IELs and DN IELs and to peripheral lymph node DN T cells that are anergic to self. What is the evidence for this assertion? In the intestine, antigen presentation to IELs bearing autoreactive TCRs fails to induce calcium mobilization, proliferation, and production of IL-2 [88]. There is also evidence that these IELs suppress inflammation [107,108]. The majority of the transgenic αβTCR^+^ DN T cells in our studies were PD-1^+^, and thus, should be anergic [33]. Furthermore, it was previously shown that transgenic TCR^+^ T cells from these double transgenic mice are very inefficient in killing H-2K^b^-bearing target cells *ex vivo* [109]. These findings are in keeping with other studies demonstrating that αβTCR^+^ DN T cells from lymph nodes of antigen-expressing mice are functionally anergic *ex vivo* [110]. Finally, the αβTCR^+^ DN T cells in the experimental model used in the Teh lab [110,111] were found to have immunosuppressive properties both *ex vivo* and *in vivo* [111]. Thus, the small number of αβ T cells bearing self-reactive TCRs and populating peripheral lymphoid tissues do not pose an immediate threat to self-tolerance and even can enforce self-tolerance.

## 13. The Early Bird Bites the Dust—The Advantages of Waiting

As discussed earlier thymocytes having TCRs that are specific for self MHC II + self-peptide and that survive negative selection become Tregs. The studies of Singer and his colleagues indicate that apoptosis of autoreactive thymocytes precedes Treg development [112,113]. They observed that differentiation of preTregs requires disruption of TCR signaling as well as the sequential upregulation of three transcription factors [112]. First cRel is expressed. This is followed by the expression of nFoxo1 and then finally the expression of Foxp3. *Ex vivo* studies [112] showed that TGF-β promotes nFoxo1 expression (presumably by disrupting TCR signal transduction). The importance of TGF-β in Foxp3^+^ preTreg development was confirmed in studies showing that the appearance of Foxp3^+^ preTregs was inhibited in TGFβRI-deficient thymocytes [112]. It remains to be discovered how TGFβ disrupts TCR signaling and whether TGFβ has other roles in promoting the differentiation of Tregs.

## 14. Me, Myself, and I

What are the specific factors in thymocyte survivors of negative selection that promote their differentiation into a tolerant cell type? As already indicated in the previous section, transcriptional control is key for the development of Tregs. IEL precursors express the transcription factor, T-bet, in response to both the cognate ligand of the TCR and the cytokine, IL-15 [114,115]. Presumably, interactions of specific TECs with the developing thymocytes promote the deployment of specific transcriptional programs. As noted earlier, surviving autoreactive DP^lo^ post-selection thymocytes give rise to PD-1^+^CCR7^+^DN and PD-1^+^CCR7^−^DN thymocytes [33]. The expression of CCR7 or lack thereof ensures that these two populations become spatially separated and complete their development in the medulla and in the cortex, respectively. There, they are exposed to different kinds of TECs and—in the case of the medulla—DCs [116,117,118,119], which provide them with the cytokines and other signals necessary for the development of their final identities.

The development of TECs is beyond the scope of this review and is beautifully presented elsewhere [120]. Here I will focus on the mature TECs—cTECs and mTECs. Many features that distinguish cTECs and mTECs have been elucidated [121]. Best understood are the differences in the antigen presentation properties of these two cell types, which make them ideally suited for their roles in positive and negative selection [122]. The “thymoproteasomes” of cTECs have three specialized subunits, β5t, β1i, and β2i. Proteasomes containing these subunits are necessary for the positive selection of thymocytes destined to become CD8^+^ T cells [123,124,125,126]. Essential to the positive selection of CD4^+^ T cells is CD83. CD83 stabilizes cell-surface MHC II molecules [127] by suppressing the ubiquitination of MHC II by the E3 ubiquitin ligase, March-1 [128]. In this way, CD83 inhibits the endocytosis of cell-surface MHC II/peptide complexes and their subsequent degradation [128].

Because of its role during negative selection in the medulla, of greater relevance to this review is the expression by mTECs of the transcription autoimmune regulator (AIRE). AIRE facilitates the expression of otherwise “tissue-restricted” antigens [129] by interacting with hypomethylated histone H3 [130] and the ATF7ip-MBD1 protein complex [131]. In so doing, AIRE influences the repertoires not only of CD4^+^ and CD8^+^ effector T cells but also of Foxp3^+^ CD4^+^ Tregs [132,133,134], which are essential for repressing autoimmunity [30,135,136]. The chromatin of mTECs is especially accessible to AIRE and to “lineage-defining” transcription factors [137,138]. Hence, chromatin structure, AIRE, and lineage-defining transcription factors work together to promote the ectopic expression of tissue-specific genes. Furthermore, AIRE also deviates autoreactive CD4^+^ thymocytes away from developing into conventional CD4^+^ T cells and to becoming Foxp3^+^ T regs [139]. Finally, AIRE appears also to have roles in homeostatic regulation of the cellular composition of the thymic medulla [140].

## 15. Meet the Great Pretenders

**Figure 6:**
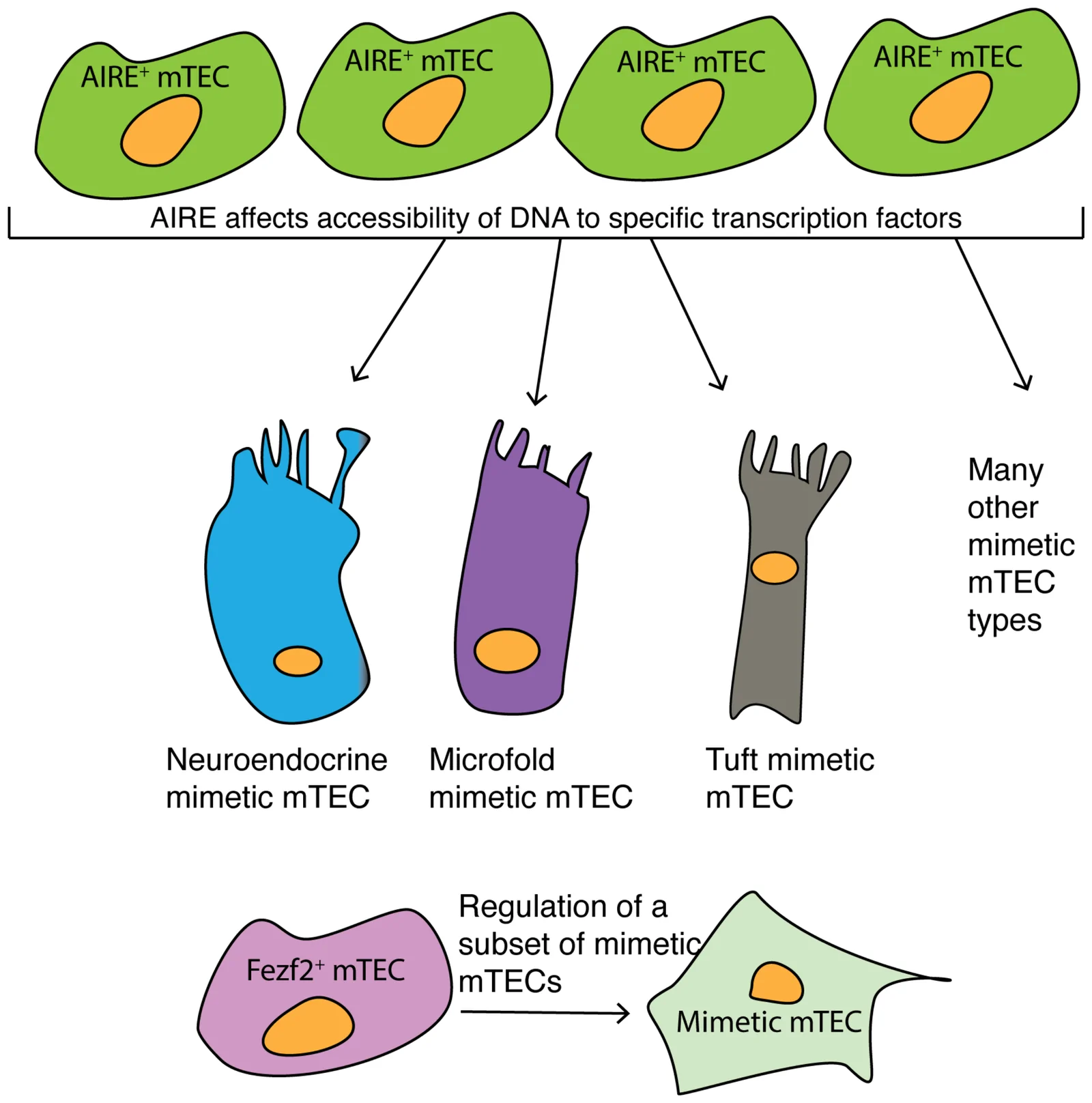
Diverse mTECs express genes of different cell lineages. As discussed in the text, AIRE promotes the accessibility of otherwise transcriptionally silent genes to tissue-specific transcription factors [147]. In contrast, Fezf2 binds to specific DNA sequences and recruits transcriptional machinery to specific genes [148]. In this regard, it acts like a canonical transcription factor.

Heretofore, I have discussed mTECs as if they are one homogeneous population. It has long been known that in fact, mTECs are heterogeneous, but the number of different types of mTECs was unknown. A game-changing breakthrough in our appreciation for the different types of mTECs came with the use of high-throughput single-cell RNA-sequencing (scRNA-seq) analysis [141,142,143,144,145]. At least thirteen types of mTECs have been identified [138,146], and the number may well increase as techniques for studying them improve. Using single-cell assay for transposase-accessible chromatin with sequencing (scATAC-seq), the Benoist and Mathis group investigated tissue-specific antigen expression in individual mTECs [138]. They confirmed that AIRE and “lineage-defining” transcription factors found in diverse tissues promote expression of a variety of tissue-specific genes. AIRE promotes chromatin accessibility at specific genes in most (but not all) mTECs. By contrast, lineage-defining transcription factors are active in distinct subsets of mTECs [138]. Each cluster is named after its cell counterpart (e.g., keratinocyte, ciliated, secretory/neuroendocrine, microfold, tuft, enterocyte/hepatocyte; Figure 6; Table 2). These mTEC are regarded as “mimetic” cells because in terms of gene expression, they mimic cell types in non-lymphoid tissues. The cell-type specific transcriptional programs of mimetic mTECs are in addition to their mTEC transcriptional profiles, so that they have a sort of dual identity [138,142].

Expression of a specific antigen by mimetic mTECs is sufficient to induce tolerance to that antigen [138]. Furthermore, mimetic cells appear to be essential for the development of antigen-specific tolerance. This was demonstrated in the case of the tuft-specific antigen, IL-25. The master regulator transcription factor Pou2f3 is required for the development of chemosensory tuft cells in the respiratory and gastrointestinal tracts and for tuft mimetic mTECs [141]. In addition, the transcription factor Insm1 is required for the development of endocrine mimetic mTECs (endoTECs) [149]. Sera from mice in which the *Insm1* gene is deleted in TECs reacted with stomach and thyroid antigens [149], indicating that endoTECs are essential for tolerance to antigens expressed by these tissues. In a third example, expression of a mimetic antigen, YFP, by specific mimetic mTECs (ciliated, muscle, or total mTECs) was found to induce tolerance to YFP and to the appearance of CD4^+^Fox3p^+^ Tregs [138].

Significantly, mimetic mTECs (first identified in mice) also occur in the human thymus [138]. Two genome-wide association studies (GWAS) studies implicated the lineage-defining transcription factors HNF4α and SPIB in inflammatory bowel disease [14,150] and primary biliary cirrhosis [151], respectively.

Another transcription factor with far-reaching effects is the previously mentioned Runx3. It plays a crucial role in mTEC development as well as in the expression of tissue-specific genes in mTECs [152]. Deletion of the *Runx3* gene in TECs causes a loss of transient amplifying cell thymic epithelial cells and of AIRE^+^ mTECs [152]. The effect on the transit-amplifying cells is notable because they give rise to AIRE^+^ mTECs [143]. Thus, the lack of AIRE^+^ mTECs in the Runx3-deficient TEC population is likely to be a downstream consequence of its absence in transit-amplifying cells. Beyond this, lack of Runx3 activity leads to drastically lower expression of tissue-specific genes in mTECs, resulting in diminished negative selection and reduced diversion of thymocytes to a Treg phenotype [152]. Mice lacking *Runx3* develop signs of autoimmune disease. In humans, *Runx3* polymorphisms correlate with risk of developing the autoimmune disease ankylosing spondylitis [153]. Also, low levels of Runx3 expression are observed in patients with systemic sclerosis [154]. Intriguingly, scRNA-seq data from human thymus tissue indicates that the *Runx3* and *Aire* genes are coordinately expressed [152].

Additionally, expression of the zinc-finger transcription factor, Ikaros, is required for AIRE^+^ mTEC development and for tissue-specific antigen gene expression [155]. Ikaros also regulates the relative proportions of mimetic mTECs and influences their gene expression patterns [155]. Unsurprisingly, ablation of *Ikzf1*, the gene encoding Ikaros, results in autoimmunity [155].

A more recently described protein that is indispensable for AIRE-independent immune tolerance is Fezf2, a Kruppel-type zinc finger protein [148]. Fezf2 interacts with the chromatin remodeler Chd4 and other proteins to directly induce the expression of tissue-specific genes [156]. Fezf2 action influences the steady state composition of a small number of mimetic mTEC populations (Figure 6), including mimetic tuft cells [157]. Interestingly, Fezf2’s actions result both in the activation of the expression of some tissue specific genes and the repression of the expression of others [156,157]. The absence of Fezf2 in TECs results in a severe reduction in mTEC expression of Pou2f3 and Hnf4a transcription factors, which are involved in the development of tuft and entero-hepato mimetic mTECs, respectively (Table 2) [138,158]. However, deletion of *Fezf2* in TECs does not lead to the loss of all mimetic mTECs [157]. Indeed, the number of mimetic mTECs was slightly elevated in mice lacking Fezf2 activity [157].

From all these studies, three major points emerge. Firstly, there are several types of mTECs, each of which contributes to the representation in the medulla of a vast swath of the gene products expressed in the various tissues of the body. Secondly, lineage-defining transcription factors are essential for medullary expression of tissue-specific genes. Thirdly, the functioning of mimetic mTECs hinges both on chromatin remodeling and on transcription factors working in concert. It is important to recall, as stated above, that some transcription factors also regulate mimetic mTEC differentiation. As already noted, Fezf2 influences the prevalence of certain mimetic mTECs. Runx3 imposes restraints on the differentiation of some mimetic mTECS [152]. Finally, as stated previously, AIRE is implicated in homeostatic regulation of the thymic medulla [140].

Given the critical roles played by CD28-, CD4-, and CD8-mediated signaling during negative selection, it is advantageous that AIRE promotes expression of CD80 and of MHC molecules by mTECs [117,159]. Thus, collectively, mTECs are armed both with MHC molecules presenting diverse self-peptides and the necessary ligands of TCR-adjacent accessory molecules to maximize the likelihood of efficient negative selection. Additionally, some mTECs produce CCL21, which recruits thymocytes and DCs to the thymic medulla [160,161,162]. Hence, together, mTECs fulfill the three critical roles essential for negative selection—the recruitment of positively selected thymocytes and other APCs (such as DCs) to the medulla, the display of MHC + self-peptides, and the expression of CD80 to maximize signal transduction through the TCR.

## 16. The More, the Better

Medullary DCs (mDCs) promote negative selection [163] and the diversion of CD4^+^ cells to a Treg phenotype. They also have been shown to mediate negative selection of MHC I-specific autoreactive thymocytes that otherwise would develop into CD8^+^ T cells [164,165]. Amongst the antigenic peptides presented by mDCs are peptides that are generated within mTECs and then subsequently are transferred to the mDCs [166]. This mTEC to mDC peptide transfer is important because of the extraordinary array of peptides generated by mimetic mTECs. Like mTECs, mDCs are heterogeneous. A recent study [167] used scRNA-seq and functional assays to characterize mDCs and found that there are seven distinct mDC subsets. These mDC subsets are functionally distinct from each other in terms of the efficiency with which they mediate negative selection versus induction of Tregs [166,167]. Together, mTECs and mDCs greatly curtail the survival of thymocytes expressing self-reactive TCRs.

## 17. Three Kisses and a Promise

The integration of signals (via the various components of their signaling machinery) from the TCR complex, from the coreceptor CD4 or CD8, and from CD28 is essential for effective silencing of autoreactive thymocytes. Failure to do so has a high cost: Any autoreactive thymocyte that escapes negative selection and fails to differentiate into a Treg (in the case of a presumptive CD4^+^ thymocyte) or into an IEL or DN T cell (in the case of a presumptive CD8^+^ thymocyte) may later cause autoimmune disease. In this regard, the fact that the various mTECs and mDC subsets have non-overlapping capabilities is particularly reassuring. These diverse sets of “self-peptide-presenting” APCs provide multiple opportunities to eliminate—or failing that—to convert an autoreactive thymocyte into a cell that promotes self-tolerance.

## 18. Future Directions—Circling Back to the Thymic Medulla as a Whole

Single-cell technologies have enabled the identification of diverse thymic medullary cells and their individual contributions to self-tolerance, which is indeed wonderful. But single-cell approaches are of limited value in determining how these cells work collectively to effectuate self-tolerance of the thymocytes that are born daily. Now, we must devise experimental systems and tools to determine how the medulla orchestrates all its individual enforcers of self-tolerance. Solving this next mystery promises to be an exciting challenge. I can hardly wait to see how it unfolds.

## Figures and Tables

**Table 1:** Fates of autoreactive thymocytes.

MHC Specificity of abTCR	Alternatively Differentiated T-Cell Precursor Type	Characteristic Marker Phenotype	References
MHC II	preTreg	CD4^+^Foxp3^+^αβTCR^+^	[28,29,30]
MHC I	CD8αα IEL precursor (type A)	CD4^−^CD8^−^CCR7^−^PD-1^+^ αβTCR^+^	[31,32]
MHC I	pre- lymph node DN T cell	CD4^−^CD8^−^CCR7^+^PD-1^+^ αβTCR^+^	[33]

**Table 2:** Expression of transcription factors by various mTECs, as determined by scRNA-seq.

	Foxn1	Trp63	Aire	Pou2f3	Spib	Sox8	Hnf4g	Hnf4a	Foxj1	Trp73	Myog	Foxi1	Foxi2	Ptf1a	Foxa2	Foxa3	Foxa1	Spdef	Grhl1	Gata3
TA	+	+	++		+															
A	+		+++		+															
T1				+	+															
T2				+++	+++															
Mi					+++	+++	+													
EH	+	+	+		+		+	+												
Ci									+++	+										
Io												+	+							
Mu											+++									
Pa														++	+		+			
Ne															++	++	++			
G																	+		+	
Bl		+															+		+	
Bs	+	+++		+															+++	++
K				++															+++	+

Data are adapted from Michelson et al. 2022 [138]. Mimetic mTECs are in the far-left column. Their identities are: TA = transit amplifying; A = Aire^+^; T1 = Tuft 1; T2 = Tuft 2; Mi = Microfold; EH = enterocyte/hepatocyte; Ci = Ciliated; Mu = Muscle; Io = Ionocyte; Pa = Ptf1a^+^ pancreatic; Ne = Neuroendocrine; G = Goblet cell; Bl = Basal (lung); Bs = Basal (skin); K = keratinocyte. The number of + symbols roughly indicates the level of expression of the given transcription factor. This representation of the data is not identical to that of Michelson et al. [138] but rather an approximate representation of it.

## Data Availability

No new data were created or analyzed in this study. Data sharing is not applicable to this article.

## Abbreviations

The following abbreviations are used in this review:

TCR	T-cell receptor for antigen
MHC	Major Histocompatibility Complex
cTEC	cortical thymic epithelial cell
APC	antigen-presenting cell
αβTCR	TCR consisting of α and β chains
DN	lacking the expression of both CD4 and CD8
DP	expressing both CD4 and CD8
SP	expressing only CD4 or only CD8
TEC	thymic epithelial cell
DC	dendritic cell
mDC	medullary dendritic cell
Treg	regulatory T cell
MOM	mitochondrial outer membrane
Apaf-1	apoptotic protease-activating factor-1
ZAP-70	Zeta-chain-associated protein kinase 70
LAT	Linker for activation of T cells
PLCγ	phospholipase Cγ
LFA-1	Lymphocyte function associated antigen 1
ICAM-1	Intercellular adhesion molecule 1
PD-1	Programmed cell death protein 1
SHP-2	Src homology 2 domain-containing phosphatase 2
IEL	Intraepithelial lymphocyte
IELp	IEL precursor
scRNA-seq	single-cell assay for transposase-accessible chromatin with sequence
endoTEC	endocrine mimetic mTEC

## References

[1] Krangel M.S. (2009). Mechanics of T cell receptor gene rearrangement. Curr. Opin. Immunol..

[2] Taniuchi I. (2018). CD4 Helper and CD8 Cytotoxic T Cell Differentiation. Annu. Rev. Immunol..

[3] Klein L., Kyewski B., Allen P.M., Hogquist K.A. (2014). Positive and negative selection of the T cell repertoire: What thymocytes see (and don’t see). Nat. Rev. Immunol..

[4] Karimi M.M., Guo Y., Cui X., Pallikonda H.A., Horková V., Wang Y.-F., Gil S.R., Rodriguez-Esteban G., Robles-Rebollo I., Bruno L. (2021). The order and logic of CD4 versus CD8 lineage choice and differentiation in mouse thymus. Nat. Commun..

[5] Shinzawa M., Moseman E.A., Gossa S., Mano Y., Bhattacharya A., Guinter T., Alag A., Chen X., Cam M., McGavern D.B. (2022). Reversal of the T cell immune system reveals the molecular basis for T cell lineage fate determination in the thymus. Nat. Immunol..

[6] He X., He X., Dave V.P., Zhang Y., Hua X., Nicolas E., Xu W., Roe B.A., Kappes D.J. (2005). The zinc finger transcription factor Th-POK regulates CD4 versus CD8 T-cell lineage commitment. Nature.

[7] Luckey M.A., Kimura M.Y., Waickman A.T., Feigenbaum L., Singer A., Park J.-H. (2014). The transcription factor ThPOK suppresses Runx3 and imposes CD4+ lineage fate by inducing the SOCS suppressors of cytokine signaling. Nat. Immunol..

[8] Sato T., Ohno S., Hayashi T., Sato C., Kohu K., Satake M., Habu S. (2005). Dual functions of Runx proteins for reactivating CD8 and silencing CD4 at the commitment process into CD8 thymocytes. Immunity.

[9] Setoguchi R., Tachibana M., Naoe Y., Muroi S., Akiyama K., Tezuka C., Okuda T., Taniuchi I. (2008). Repression of the transcription factor Th-POK by Runx complexes in cytotoxic T cell development. Science.

[10] Taniuchi I., Osato M., Egawa T., Sunshine M.J., Bae S.C., Komori T., Ito Y., Littman D.R. (2002). Differential requirements for Runx proteins in CD4 repression and epigenetic silencing during T lymphocyte development. Cell.

[11] Egawa T., Tillman R.E., Naoe Y., Taniuchi I., Littman D.R. (2007). The role of the Runx transcription factors in thymocyte differentiation and in homeostasis of naive T cells. J. Exp. Med..

[12] Steier Z., Kim E.J.Y., Aylard D.A., Robey E.A. (2024). The CD4 Versus CD8 T Cell Fate Decision: A Multiomics-Informed Perspective. Annu. Rev. Immunol..

[13] Esensten J.H., Helou Y.A., Chopra G., Weiss A., Bluestone J.A. (2016). CD28 Costimulation: From Mechanism to Therapy. Immunity.

[14] Gmünder H., Lesslauer W. (1984). A 45-kDa human T-cell membrane glycoprotein functions in the regulation of cell proliferative responses. Eur. J. Biochem..

[15] Damle N.K., Doyle L.V., Grosmaire L.S., Ledbetter J.A. (1988). Differential regulatory signals delivered by antibody binding to the CD28 (Tp44) molecule during the activation of human T lymphocytes. J. Immunol..

[16] Damle N.K., Doyle L.V. (1989). Stimulation via the CD3 and CD28 molecules induces responsiveness to IL-4 in CD4+CD29+CD45R- memory T lymphocytes. J. Immunol..

[17] Gross J.A., St John T., Allison J.P. (1990). The murine homologue of the T lymphocyte antigen CD28. Molecular cloning and cell surface expression. J. Immunol..

[18] Shahinian A., Pfeffer K., Lee K.P., Kündig T.M., Kishihara K., Wakeham A., Kawai K., Ohashi P.S., Thompson C.B., Mak T.W. (1993). Differential T cell costimulatory requirements in CD28-deficient mice. Science.

[19] Bachmann M.F., Sebzda E., Kündig T.M., Shahinian A., Speiser D.E., Mak T.W., Ohashi P.S. (1996). T cell responses are governed by avidity and co-stimulatory thresholds. Eur. J. Immunol..

[20] Boomer J.S., Green J.M. (2010). An enigmatic tail of CD28 signaling. Cold Spring Harb. Perspect. Biol..

[21] Bachmann M.F., McKall-Faienza K., Schmits R., Bouchard D., Beach J., Speiser D.E., Mak T.W., Ohashi P.S. (1997). Distinct roles for LFA-1 and CD28 during activation of naive T cells: Adhesion versus costimulation. Immunity.

[22] Gross J.A., Callas E., Allison J.P. (1992). Identification and distribution of the costimulatory receptor CD28 in the mouse. J. Immunol..

[23] Inaba K., Witmer-Pack M., Inaba M., Hathcock K.S., Sakuta H., Azuma M., Yagita H., Okumura K., Linsley P.S., Ikehara S. (1994). The tissue distribution of the B7-2 costimulator in mice: Abundant expression on dendritic cells in situ and during maturation *in vitro*. J. Exp. Med..

[24] Reiser H., Schneeberger E.E. (1994). The costimulatory molecule B7 is expressed in the medullary region of the murine thymus. Immunology.

[25] Zuklys S., Balciunaite G., Agarwal A., Fasler-Kan E., Palmer E., Holländer G.A. (2000). Normal thymic architecture and negative selection are associated with Aire expression, the gene defective in the autoimmune-polyendocrinopathy-candidiasis-ectodermal dystrophy (APECED). J. Immunol..

[26] Ueno T., Saito F., Gray D.H.D., Kuse S., Hieshima K., Nakano H., Kakiuchi T., Lipp M., Boyd R.L., Takahama Y. (2004). CCR7 signals are essential for cortex-medulla migration of developing thymocytes. J. Exp. Med..

[27] Griffith A.V., Fallahi M., Nakase H., Gosink M., Young B., Petrie H.T. (2009). Spatial mapping of thymic stromal microenvironments reveals unique features influencing T lymphoid differentiation. Immunity.

[28] Sakaguchi S., Yamaguchi T., Nomura T., Ono M. (2008). Regulatory T cells and immune tolerance. Cell.

[29] Sakaguchi S., Wing K., Onishi Y., Prieto-Martin P., Yamaguchi T. (2009). Regulatory T cells: How do they suppress immune responses?. Int. Immunol..

[30] Wing K., Sakaguchi S. (2010). Regulatory T cells exert checks and balances on self tolerance and autoimmunity. Nat. Immunol..

[31] Golec D.P., Hoeppli R.E., Henao Caviedes L.M., McCann J., Levings M.K., Baldwin T.A. (2017). Thymic progenitors of TCRαβ. J. Exp. Med..

[32] Ruscher R., Kummer R.L., Lee Y.J., Jameson S.C., Hogquist K.A. (2017). CD8αα intraepithelial lymphocytes arise from two main thymic precursors. Nat. Immunol..

[33] Zúñiga M.C., Hyun S., Du J., Dubiner S., Freedman-Goretsky A., Goretsky Y., Pothukuchi A., Fong N.A.Y., Berg A., Davis C. (2026). Fine tuning of TCR signaling via CD8αβ and PD-1 and the fate of autoreactive thymocytes during negative selection. Immunohorizons.

[34] Nielsen H.V., Yang L., Mueller J.L., Ritter A.J., Hiwa R., Proekt I., Rackaityte E., Aylard D., Gupta M., Scharer C.D. (2025). Nr4a1 and Nr4a3 redundantly control clonal deletion and contribute to an anergy-like transcriptome in auto-reactive thymocytes to impose tolerance in mice. Nat. Commun..

[35] Pang Y.-P., Dai H., Smith A., Meng X.W., Schneider P.A., Kaufmann S.H. (2012). Bak Conformational Changes Induced by Ligand Binding: Insight into BH3 Domain Binding and Bak Homo-Oligomerization. Sci. Rep..

[36] Moldoveanu T., Czabotar P.E. (2020). BAX, BAK, and BOK: A Coming of Age for the BCL-2 Family Effector Proteins. Cold Spring Harb. Perspect. Biol..

[37] Yuan S., Akey C.W. (2013). Apoptosome structure, assembly, and procaspase activation. Structure.

[38] Huang D.C., Strasser A. (2000). BH3-Only proteins-essential initiators of apoptotic cell death. Cell.

[39] Strasser A. (2005). The role of BH3-only proteins in the immune system. Nat. Rev. Immunol..

[40] Liu X., Dai S., Zhu Y., Marrack P., Kappler J.W. (2003). The structure of a Bcl-xL/Bim fragment complex: Implications for Bim function. Immunity.

[41] Bouillet P., Purton J.F., Godfrey D.I., Zhang L.-C., Coultas L., Puthalakath H., Pellegrini M., Cory S., Adams J.M., Strasser A. (2002). BH3-only Bcl-2 family member Bim is required for apoptosis of autoreactive thymocytes. Nature.

[42] Suen A.Y.W., Baldwin T.A. (2012). Proapoptotic protein Bim is differentially required during thymic clonal deletion to ubiquitous versus tissue-restricted antigens. Proc. Natl. Acad. Sci. USA.

[43] Gray D.H.D., Kupresanin F., Berzins S.P., Herold M.J., O’Reilly L.A., Bouillet P., Strasser A. (2012). The BH3-only proteins Bim and Puma cooperate to impose deletional tolerance of organ-specific antigens. Immunity.

[44] Chipuk J.E., Green D.R. (2009). PUMA cooperates with direct activator proteins to promote mitochondrial outer membrane permeabilization and apoptosis. Cell Cycle.

[45] Kim P.W., Sun Z.-Y.J., Blacklow S.C., Wagner G., Eck M.J. (2003). A zinc clasp structure tethers Lck to T cell coreceptors CD4 and CD8. Science.

[46] Woessner N.M., Uleri V., Stepanek O., Minguet S. (2025). The TCR and LCK: Foundations for T-cell activation and therapeutic innovation. Front. Immunol..

[47] Kabouridis P.S., Magee A.I., Ley S.C. (1997). S-acylation of LCK protein tyrosine kinase is essential for its signalling function in T lymphocytes. EMBO J..

[48] Zhang W., Sloan-Lancaster J., Kitchen J., Trible R.P., Samelson L.E. (1998). LAT: The ZAP-70 tyrosine kinase substrate that links T cell receptor to cellular activation. Cell.

[49] Bosselut R., Kubo S., Guinter T., Kopacz J.L., Altman J.D., Feigenbaum L., Singer A. (2000). Role of CD8beta domains in CD8 coreceptor function: Importance for MHC I binding, signaling, and positive selection of CD8+ T cells in the thymus. Immunity.

[50] Bi K., Tanaka Y., Coudronniere N., Sugie K., Hong S., van Stipdonk M.J., Altman A. (2001). Antigen-induced translocation of PKC-theta to membrane rafts is required for T cell activation. Nat. Immunol..

[51] Verí M.C., DeBell K.E., Seminario M.C., DiBaldassarre A., Reischl I., Rawat R., Graham L., Noviello C., Rellahan B.L., Miscia S. (2001). Membrane raft-dependent regulation of phospholipase Cgamma-1 activation in T lymphocytes. Mol. Cell. Biol..

[52] Shi H., Shao B. (2023). LFA-1 Activation in T-Cell Migration and Immunological Synapse Formation. Cells.

[53] Springer T.A., Dustin M.L. (2012). Integrin inside-out signaling and the immunological synapse. Curr. Opin. Cell Biol..

[54] Zamoyska R. (1994). The CD8 coreceptor revisited: One chain good, two chains better. Immunity.

[55] Renard V., Romero P., Vivier E., Malissen B., Luescher I.F. (1996). CD8 beta increases CD8 coreceptor function and participation in TCR-ligand binding. J. Exp. Med..

[56] Witte T., Spoerl R., Chang H.C. (1999). The CD8beta ectodomain contributes to the augmented coreceptor function of CD8alphabeta heterodimers relative to CD8alphaalpha homodimers. Cell. Immunol..

[57] Turner J.M., Brodsky M.H., Irving B.A., Levin S.D., Perlmutter R.M., Littman D.R. (1990). Interaction of the unique N-terminal region of tyrosine kinase p56lck with cytoplasmic domains of CD4 and CD8 is mediated by cysteine motifs. Cell.

[58] Horkova V., Drobek A., Mueller D., Gubser C., Niederlova V., Wyss L., King C.G., Zehn D., Stepanek O. (2020). Dynamics of the Coreceptor-LCK Interactions during T Cell Development Shape the Self-Reactivity of Peripheral CD4 and CD8 T Cells. Cell Rep..

[59] Watanabe M., Lu Y., Breen M., Hodes R.J. (2020). B7-CD28 co-stimulation modulates central tolerance via thymic clonal deletion and Treg generation through distinct mechanisms. Nat. Commun..

[60] Teh H.S., Teh S.J. (2001). The affinity/avidity and length of exposure to the deleting ligand determine dependence on CD28 for the efficient deletion of self-specific CD4+CD8+ thymocytes. Cell. Immunol..

[61] Dustin M.L., Groves J.T. (2012). Receptor signaling clusters in the immune synapse. Annu. Rev. Biophys..

[62] Filipp D., Ballek O., Manning J. (2012). Lck, Membrane Microdomains, and TCR Triggering Machinery: Defining the New Rules of Engagement. Front. Immunol..

[63] Simons K., van Meer G. (1988). Lipid sorting in epithelial cells. Biochemistry.

[64] Harder T., Kuhn M. (2000). Selective Accumulation of Raft-Associated Membrane Protein Lat in T Cell Receptor Signaling Assemblies. J. Cell Biol..

[65] Zeyda M., Staffler G., Hořejší V., Waldhäusl W., Stulnig T.M. (2002). LAT Displacement from Lipid Rafts as a Molecular Mechanism for the Inhibition of T Cell Signaling by Polyunsaturated Fatty Acids. J. Biol. Chem..

[66] Swamy M., Beck-Garcia K., Beck-Garcia E., Hartl F.A., Morath A., Yousefi O.S., Dopfer E.P., Molnár E., Schulze A.K., Blanco R. (2016). A Cholesterol-Based Allostery Model of T Cell Receptor Phosphorylation. Immunity.

[67] Chen Y., Zhu Y., Li X., Gao W., Zhen Z., Dong D., Huang B., Ma Z., Zhang A., Song X. (2022). Cholesterol inhibits TCR signaling by directly restricting TCR-CD3 core tunnel motility. Mol. Cell.

[68] Martin M., Schneider H., Azouz A., Rudd C.E. (2001). Cytotoxic T lymphocyte antigen 4 and CD28 modulate cell surface raft expression in their regulation of T cell function. J. Exp. Med..

[69] Pang D.J., Hayday A.C., Bijlmakers M.-J. (2007). CD8 Raft localization is induced by its assembly into CD8alpha beta heterodimers, Not CD8alpha alpha homodimers. J. Biol. Chem..

[70] Arcaro A., Grégoire C., Boucheron N., Stotz S., Palmer E., Malissen B., Luescher I.F. (2000). Essential role of CD8 palmitoylation in CD8 coreceptor function. J. Immunol..

[71] Arcaro A., Grégoire C., Bakker T.R., Baldi L., Jordan M., Goffin L., Boucheron N., Wurm F., van der Merwe P.A., Malissen B. (2001). CD8beta endows CD8 with efficient coreceptor function by coupling T cell receptor/CD3 to raft-associated CD8/p56(lck) complexes. J. Exp. Med..

[72] Crooks M.E., Littman D.R. (1994). Disruption of T lymphocyte positive and negative selection in mice lacking the CD8 beta chain. Immunity.

[73] Irie H.Y., Mong M.S., Itano A., Crooks M.E., Littman D.R., Burakoff S.J., Robey E. (1998). The cytoplasmic domain of CD8 beta regulates Lck kinase activation and CD8 T cell development. J. Immunol..

[74] Nakayama K., Nakayama K., Negishi I., Kuida K., Louie M.C., Kanagawa O., Nakauchi H., Loh D.Y. (1994). Requirement for CD8 beta chain in positive selection of CD8-lineage T cells. Science.

[75] Barnden M.J., Heath W.R., Carbone F.R. (1997). Down-modulation of CD8 beta-chain in response to an altered peptide ligand enables developing thymocytes to escape negative selection. Cell. Immunol..

[76] Chidgey A., Boyd R. (1997). Agonist peptide modulates T cell selection thresholds through qualitative and quantitative shifts in CD8 co-receptor expression. Int. Immunol..

[77] Banani S.F., Lee H.O., Hyman A.A., Rosen M.K. (2017). Biomolecular condensates: Organizers of cellular biochemistry. Nat. Rev. Mol. Cell Biol..

[78] Banjade S., Rosen M.K. (2014). Phase transitions of multivalent proteins can promote clustering of membrane receptors. eLife.

[79] Su X., Ditlev J.A., Hui E., Xing W., Banjade S., Okrut J., King D.S., Taunton J., Rosen M.K., Vale R.D. (2016). Phase separation of signaling molecules promotes T cell receptor signal transduction. Science.

[80] Wang H.-Y., Chan S.H., Dey S., Castello-Serrano I., Rosen M.K., Ditlev J.A., Levental K.R., Levental I. (2023). Coupling of protein condensates to ordered lipid domains determines functional membrane organization. Sci. Adv..

[81] McKeithan T.W. (1995). Kinetic proofreading in T-cell receptor signal transduction. Proc. Natl. Acad. Sci. USA.

[82] Lo W.-L., Weiss A. (2021). Adapting T Cell Receptor Ligand Discrimination Capability via LAT. Front. Immunol..

[83] Au-Yeung B.B., Deindl S., Hsu L.-Y., Palacios E.H., Levin S.E., Kuriyan J., Weiss A. (2009). The structure, regulation, and function of ZAP-70. Immunol. Rev..

[84] Gangopadhyay K., Manna B., Roy S., Kumari S., Debnath O., Chowdhury S., Ghosh A., Das R. (2020). An allosteric hot spot in the tandem-SH2 domain of ZAP-70 regulates T-cell signaling. Biochem. J..

[85] Leishman A.J., Gapin L., Capone M., Palmer E., MacDonald H.R., Kronenberg M., Cheroutre H. (2002). Precursors of Functional MHC Class I- or Class II-Restricted CD8αα+ T Cells Are Positively Selected in the Thymus by Agonist Self-Peptides. Immunity.

[86] McDonald B.D., Bunker J.J., Ishizuka I.E., Jabri B., Bendelac A. (2014). Elevated T cell receptor signaling identifies a thymic precursor to the TCRαβ(+)CD4(-)CD8β(-) intraepithelial lymphocyte lineage. Immunity.

[87] Mayans S., Stepniak D., Palida S., Larange A., Dreux J., Arlian B., Shinnakasu R., Kronenberg M., Cheroutre H., Lambolez F. (2014). αβT cell receptors expressed by CD4(-)CD8αβ(-) intraepithelial T cells drive their fate into a unique lineage with unusual MHC reactivities. Immunity.

[88] Pobezinsky L.A., Angelov G.S., Tai X., Jeurling S., Van Laethem F., Feigenbaum L., Park J.-H., Singer A. (2012). Clonal deletion and the fate of autoreactive thymocytes that survive negative selection. Nat. Immunol..

[89] Kurd N.S., Hoover A., Yoon J., Weist B.M., Lutes L., Chan S.W., Robey E.A. (2021). Factors that influence the thymic selection of CD8αα intraepithelial lymphocytes. Mucosal Immunol..

[90] McGargill M.A., Hogquist K.A. (1999). Antigen-induced coreceptor down-regulation on thymocytes is not a result of apoptosis. J. Immunol..

[91] Ishida Y., Agata Y., Shibahara K., Honjo T. (1992). Induced expression of PD-1, a novel member of the immunoglobulin gene superfamily, upon programmed cell death. EMBO J..

[92] Freeman G.J., Long A.J., Iwai Y., Bourque K., Chernova T., Nishimura H., Fitz L.J., Malenkovich N., Okazaki T., Byrne M.C. (2000). Engagement of the PD-1 immunoinhibitory receptor by a novel B7 family member leads to negative regulation of lymphocyte activation. J. Exp. Med..

[93] Parry R.V., Chemnitz J.M., Frauwirth K.A., Lanfranco A.R., Braunstein I., Kobayashi S.V., Linsley P.S., Thompson C.B., Riley J.L. (2005). CTLA-4 and PD-1 receptors inhibit T-cell activation by distinct mechanisms. Mol. Cell. Biol..

[94] Keir M.E., Latchman Y.E., Freeman G.J., Sharpe A.H. (2005). Programmed death-1 (PD-1):PD-ligand 1 interactions inhibit TCR-mediated positive selection of thymocytes. J. Immunol..

[95] Sheppard K.-A., Fitz L.J., Lee J.M., Benander C., George J.A., Wooters J., Qiu Y., Jussif J.M., Carter L.L., Wood C.R. (2004). PD-1 inhibits T-cell receptor induced phosphorylation of the ZAP70/CD3zeta signalosome and downstream signaling to PKCtheta. FEBS Lett..

[96] Yokosuka T., Takamatsu M., Kobayashi-Imanishi W., Hashimoto-Tane A., Azuma M., Saito T. (2012). Programmed cell death 1 forms negative costimulatory microclusters that directly inhibit T cell receptor signaling by recruiting phosphatase SHP2. J. Exp. Med..

[97] Mizuno R., Sugiura D., Shimizu K., Maruhashi T., Watada M., Okazaki I.-M., Okazaki T. (2019). PD-1 Primarily Targets TCR Signal in the Inhibition of Functional T Cell Activation. Front. Immunol..

[98] White D., Cote-Martin A., Bleck M., Garaffa N., Shaaban A., Wu H., Liu D., Young D., Scheer J., Lorenz I.C. (2023). Programmed Cell Death-1 (PD-1) anchoring to the GPI-linked co-receptor CD48 reveals a novel mechanism to modulate PD-1-dependent inhibition of human T cells. Mol. Immunol..

[99] Pralle A., Keller P., Florin E.L., Simons K., Hörber J.K. (2000). Sphingolipid-cholesterol rafts diffuse as small entities in the plasma membrane of mammalian cells. J. Cell Biol..

[100] Simons K., Toomre D. (2000). Lipid rafts and signal transduction. Nat. Rev. Mol. Cell Biol..

[101] Hui E., Cheung J., Zhu J., Su X., Taylor M.J., Wallweber H.A., Sasmal D.K., Huang J., Kim J.M., Mellman I. (2017). T cell costimulatory receptor CD28 is a primary target for PD-1-mediated inhibition. Science.

[102] Wang Q., Bardhan K., Boussiotis V.A., Patsoukis N. (2021). The PD-1 Interactome. Adv. Biol..

[103] Harder T., Scheiffele P., Verkade P., Simons K. (1998). Lipid domain structure of the plasma membrane revealed by patching of membrane components. J. Cell Biol..

[104] Janes P.W., Ley S.C., Magee A.I. (1999). Aggregation of lipid rafts accompanies signaling via the T cell antigen receptor. J. Cell Biol..

[105] Gangadharan D., Lambolez F., Attinger A., Wang-Zhu Y., Sullivan B.A., Cheroutre H. (2006). Identification of pre- and postselection TCRalphabeta+ intraepithelial lymphocyte precursors in the thymus. Immunity.

[106] Guy-Grand D., Cerf-Bensussan N., Malissen B., Malassis-Seris M., Briottet C., Vassalli P. (1991). Two gut intraepithelial CD8+ lymphocyte populations with different T cell receptors: A role for the gut epithelium in T cell differentiation. J. Exp. Med..

[107] Denning T.L., Granger S.W., Mucida D., Graddy R., Leclercq G., Zhang W., Honey K., Rasmussen J.P., Cheroutre H., Rudensky A.Y. (2007). Mouse TCRalphabeta+CD8alphaalpha intraepithelial lymphocytes express genes that down-regulate their antigen reactivity and suppress immune responses. J. Immunol..

[108] Peterson L.W., Artis D. (2014). Intestinal epithelial cells: Regulators of barrier function and immune homeostasis. Nat. Rev. Immunol..

[109] Sponaas A.M., Tomlinson P.D., Schulz R., Antoniou J., Ize-Iyamu P., Schmitt-Verhulst A.M., Mellor A.L. (1994). Tolerance induction by elimination of subsets of self-reactive thymocytes. Int. Immunol..

[110] Caveno J., Zhang Y., Motyka B., Teh S.J., Teh H.S. (1999). Functional similarity and differences between selection-independent CD4-CD8- alphabeta T cells and positively selected CD8 T cells expressing the same TCR and the induction of anergy in CD4-CD8- alphabeta T cells in antigen-expressing mice. J. Immunol..

[111] Priatel J.J., Utting O., Teh H.S. (2001). TCR/self-antigen interactions drive double-negative T cell peripheral expansion and differentiation into suppressor cells. J. Immunol..

[112] Tai X., Indart A., Rojano M., Guo J., Apenes N., Kadakia T., Craveiro M., Alag A., Etzensperger R., Badr M.E. (2023). How autoreactive thymocytes differentiate into regulatory versus effector CD4+ T cells after avoiding clonal deletion. Nat. Immunol..

[113] Tai X., Singer A. (2024). New insights into developmental fate decisions by autoreactive CD4 thymocytes. Clin. Transl. Med..

[114] Klose C.S.N., Blatz K., d’Hargues Y., Hernandez P.P., Kofoed-Nielsen M., Ripka J.F., Ebert K., Arnold S.J., Diefenbach A., Palmer E. (2014). The transcription factor T-bet is induced by IL-15 and thymic agonist selection and controls CD8αα(+) intraepithelial lymphocyte development. Immunity.

[115] Klose C.S.N., Hummel J.F., Faller L., d’Hargues Y., Ebert K., Tanriver Y. (2018). A committed postselection precursor to natural TCRαβ+ intraepithelial lymphocytes. Mucosal Immunol..

[116] Nita A., Nishiyama M., Muto Y., Nakayama K.I. (2016). FBXL12 regulates T-cell differentiation in a cell-autonomous manner. Genes. Cells.

[117] Irla M. (2025). Thymic Crosstalk: An Overview of the Complex Cellular Interactions that Control the Establishment of T-Cell Tolerance. Adv. Exp. Med. Biol..

[118] Kurd N., Robey E.A. (2016). T-cell selection in the thymus: A spatial and temporal perspective. Immunol. Rev..

[119] Vobořil M., Xuan S., Hogquist K.A. (2025). Thymic Dendritic Cells Revisited. Immunol. Rev..

[120] Anderson G., Cosway E.J., James K.D., Ohigashi I., Takahama Y. (2024). Generation and repair of thymic epithelial cells. J. Exp. Med..

[121] Anderson G., Takahama Y. (2012). Thymic epithelial cells: Working class heroes for T cell development and repertoire selection. Trends Immunol..

[122] Klein L., Petrozziello E. (2025). Antigen presentation for central tolerance induction. Nat. Rev. Immunol..

[123] Murata S., Sasaki K., Kishimoto T., Niwa S.-I., Hayashi H., Takahama Y., Tanaka K. (2007). Regulation of CD8+ T cell development by thymus-specific proteasomes. Science.

[124] Nitta T., Murata S., Sasaki K., Fujii H., Ripen A.M., Ishimaru N., Koyasu S., Tanaka K., Takahama Y. (2010). Thymoproteasome shapes immunocompetent repertoire of CD8+ T cells. Immunity.

[125] Takada K., Van Laethem F., Xing Y., Akane K., Suzuki H., Murata S., Tanaka K., Jameson S.C., Singer A., Takahama Y. (2015). TCR affinity for thymoproteasome-dependent positively selecting peptides conditions antigen responsiveness in CD8(+) T cells. Nat. Immunol..

[126] Sasaki K., Takada K., Ohte Y., Kondo H., Sorimachi H., Tanaka K., Takahama Y., Murata S. (2015). Thymoproteasomes produce unique peptide motifs for positive selection of CD8(+) T cells. Nat. Commun..

[127] von Rohrscheidt J., Petrozziello E., Nedjic J., Federle C., Krzyzak L., Ploegh H.L., Ishido S., Steinkasserer A., Klein L. (2016). Thymic CD4 T cell selection requires attenuation of March8-mediated MHCII turnover in cortical epithelial cells through CD83. J. Exp. Med..

[128] Kaul S., Cole N.B., Bandola-Simon J., Lu Y., Tondini E., Walseng E., Roche P.A. (2025). CD83 suppresses endogenous March-I-dependent MHC class II ubiquitination, endocytosis, and degradation. Proc. Natl. Acad. Sci. USA.

[129] Anderson M.S., Venanzi E.S., Klein L., Chen Z., Berzins S.P., Turley S.J., von Boehmer H., Bronson R., Dierich A., Benoist C. (2002). Projection of an immunological self shadow within the thymus by the aire protein. Science.

[130] Koh A.S., Kingston R.E., Benoist C., Mathis D. (2010). Global relevance of Aire binding to hypomethylated lysine-4 of histone-3. Proc. Natl. Acad. Sci. USA.

[131] Waterfield M., Khan I.S., Cortez J.T., Fan U., Metzger T., Greer A., Fasano K., Martinez-Llordella M., Pollack J.L., Erle D.J. (2014). The transcriptional regulator Aire coopts the repressive ATF7ip-MBD1 complex for the induction of immunotolerance. Nat. Immunol..

[132] Peterson P., Org T., Rebane A. (2008). Transcriptional regulation by AIRE: Molecular mechanisms of central tolerance. Nat. Rev. Immunol..

[133] Speck-Hernandez C.A., Assis A.F., Felicio R.F., Cotrim-Sousa L., Pezzi N., Lopes G.S., Bombonato-Prado K.F., Giuliatti S., Passos G.A. (2018). Aire Disruption Influences the Medullary Thymic Epithelial Cell Transcriptome and Interaction With Thymocytes. Front. Immunol..

[134] Passos G.A., Speck-Hernandez C.A., Assis A.F., Mendes-da-Cruz D.A. (2018). Update on Aire and thymic negative selection. Immunology.

[135] Hsieh C.-S., Lee H.-M., Lio C.-W.J. (2012). Selection of regulatory T cells in the thymus. Nat. Rev. Immunol..

[136] Josefowicz S.Z., Lu L.-F., Rudensky A.Y. (2012). Regulatory T cells: Mechanisms of differentiation and function. Annu. Rev. Immunol..

[137] Guerau-de-Arellano M., Martinic M., Benoist C., Mathis D. (2009). Neonatal tolerance revisited: A perinatal window for Aire control of autoimmunity. J. Exp. Med..

[138] Michelson D.A., Hase K., Kaisho T., Benoist C., Mathis D. (2022). Thymic epithelial cells co-opt lineage-defining transcription factors to eliminate autoreactive T cells. Cell.

[139] Malchow S., Leventhal D.S., Lee V., Nishi S., Socci N.D., Savage P.A. (2016). Aire Enforces Immune Tolerance by Directing Autoreactive T Cells into the Regulatory T Cell Lineage. Immunity.

[140] Peterson P. (2026). AIRE’s Complex Role Beyond Promiscuous Gene Expression. Immunol. Rev..

[141] Bornstein C., Nevo S., Giladi A., Kadouri N., Pouzolles M., Gerbe F., David E., Machado A., Chuprin A., Tóth B. (2018). Single-cell mapping of the thymic stroma identifies IL-25-producing tuft epithelial cells. Nature.

[142] Miller C.N., Proekt I., von Moltke J., Wells K.L., Rajpurkar A.R., Wang H., Rattay K., Khan I.S., Metzger T.C., Pollack J.L. (2018). Thymic tuft cells promote an IL-4-enriched medulla and shape thymocyte development. Nature.

[143] Wells K.L., Miller C.N., Gschwind A.R., Wei W., Phipps J.D., Anderson M.S., Steinmetz L.M. (2020). Combined transient ablation and single-cell RNA-sequencing reveals the development of medullary thymic epithelial cells. eLife.

[144] Miyao T., Miyauchi M., Kelly S.T., Terooatea T.W., Ishikawa T., Oh E., Hirai S., Horie K., Takakura Y., Ohki H. (2022). Integrative analysis of scRNA-seq and scATAC-seq revealed transit-amplifying thymic epithelial cells expressing autoimmune regulator. eLife.

[145] Matsumoto M., Yoshida H., Tsuneyama K., Oya T., Matsumoto M. (2023). Revisiting Aire and tissue-restricted antigens at single-cell resolution. Front. Immunol..

[146] Muro R., Nitta T. (2026). Breaking the upper limit of mTEC heterogeneity. Nat. Immunol..

[147] Giraud M., Peterson P. (2025). The Autoimmune Regulator (AIRE) Gene, The Master Activator of Self-Antigen Expression in the Thymus. Adv. Exp. Med. Biol..

[148] Takaba H., Morishita Y., Tomofuji Y., Danks L., Nitta T., Komatsu N., Kodama T., Takayanagi H. (2015). Fezf2 Orchestrates a Thymic Program of Self-Antigen Expression for Immune Tolerance. Cell.

[149] Givony T., Leshkowitz D., Del Castillo D., Nevo S., Kadouri N., Dassa B., Gruper Y., Khalaila R., Ben-Nun O., Gome T. (2023). Thymic mimetic cells function beyond self-tolerance. Nature.

[150] Barrett J.C., Lee J.C., Lees C.W., Prescott N.J., Anderson C.A., Phillips A., Wesley E., Parnell K., Zhang H., UK IBD Genetics Consortium (2009). Genome-wide association study of ulcerative colitis identifies three new susceptibility loci, including the HNF4A region. Nat. Genet..

[151] Liu X., Invernizzi P., Lu Y., Kosoy R., Lu Y., Bianchi I., Podda M., Xu C., Xie G., Macciardi F. (2010). Genome-wide meta-analyses identify three loci associated with primary biliary cirrhosis. Nat. Genet..

[152] Sin J.H., Phillips S.H., Parent A.V., Waterfield M.R. (2026). Runx3 instructs Aire+ mTEC development, TSA gene expression, and central tolerance. J. Autoimmun..

[153] Liu H., Fu L., He D., Deng J., Zhu J., Xu K., Hu D., Li J., Wang Y., Hu W. (2020). RUNX3 Polymorphisms Affect the Risk of Ankylosing Spondylitis. Med. Sci. Monit..

[154] Affandi A.J., Carvalheiro T., Ottria A., Broen J.C., Bossini-Castillo L., Tieland R.G., van Bon L., Chouri E., Rossato M., Mertens J.S. (2019). Low RUNX3 expression alters dendritic cell function in patients with systemic sclerosis and contributes to enhanced fibrosis. Ann. Rheum. Dis..

[155] Sin J.H., Sucharov J., Kashyap S., Wang Y., Proekt I., Liu X., Parent A.V., Gupta A., Kastner P., Chan S. (2023). Ikaros is a principal regulator of Aire+ mTEC homeostasis, thymic mimetic cell diversity, and central tolerance. Sci. Immunol..

[156] Tomofuji Y., Takaba H., Suzuki H.I., Benlaribi R., Martinez C.D.P., Abe Y., Morishita Y., Okamura T., Taguchi A., Kodama T. (2020). Chd4 choreographs self-antigen expression for central immune tolerance. Nat. Immunol..

[157] Ushio A., Matsuda-Lennikov M., Kalle-Youngoue F., Shimizu A., Abdelmaksoud A., Kelly M.C., Ishimaru N., Takahama Y. (2024). Functionally diverse thymic medullary epithelial cells interplay to direct central tolerance. Cell Rep..

[158] Lammers S., Barrera V., Brennecke P., Miller C., Yoon J., Balolong J., Anderson M.S., Ho Sui S., Steinmetz L.M., von Andrian U.H. (2023). Ehf and Fezf2 regulate late medullary thymic epithelial cell and thymic tuft cell development. Front. Immunol..

[159] Gäbler J., Arnold J., Kyewski B. (2007). Promiscuous gene expression and the developmental dynamics of medullary thymic epithelial cells. Eur. J. Immunol..

[160] Ohigashi I., Kyuma H., Otsu E., Hayashi S., Takemoto T., Takahama Y. (2025). Ccl21a, Rather Than Ccl21b, is Essential for Thymocyte Migration in Mouse. Eur. J. Immunol..

[161] Kozai M., Kubo Y., Katakai T., Kondo H., Kiyonari H., Schaeuble K., Luther S.A., Ishimaru N., Ohigashi I., Takahama Y. (2017). Essential role of CCL21 in establishment of central self-tolerance in T cells. J. Exp. Med..

[162] Michalaki C., Sharjeel M., Cowan J.E. (2026). From Development to Aging: Dynamic Roles of the Thymic Medulla in T Cell Biology. Immunol. Rev..

[163] Ohnmacht C., Pullner A., King S.B.S., Drexler I., Meier S., Brocker T., Voehringer D. (2009). Constitutive ablation of dendritic cells breaks self-tolerance of CD4 T cells and results in spontaneous fatal autoimmunity. J. Exp. Med..

[164] Li H., Hsu H.-C., Wu Q., Yang P., Li J., Luo B., Oukka M., Steele C.H., Cua D.J., Grizzle W.E. (2014). IL-23 promotes TCR-mediated negative selection of thymocytes through the upregulation of IL-23 receptor and RORγt. Nat. Commun..

[165] Wu L., Shortman K. (2005). Heterogeneity of thymic dendritic cells. Semin. Immunol..

[166] Březina J., Vobořil M., Filipp D. (2022). Mechanisms of Direct and Indirect Presentation of Self-Antigens in the Thymus. Front. Immunol..

[167] Srinivasan J., Moore C.R., Calindi A., Helm B.R., Yang Y., Moore J.F., Selden H.J., Heiser C.N., Liu Q., Lau K.S. (2026). The differentiation and function of heterogeneous thymic dendritic cell subsets require signals provided by distinct thymocyte cell types. Nat. Immunol..

